# Suggestions for extending the FAIR Principles based on a linguistic perspective on semantic interoperability

**DOI:** 10.1038/s41597-025-05011-x

**Published:** 2025-04-24

**Authors:** Lars Vogt, Philip Strömert, Nicolas Matentzoglu, Naouel Karam, Marcel Konrad, Manuel Prinz, Roman Baum

**Affiliations:** 1 TIB Leibniz Information Centre for Science and Technology, Welfengarten 1B, 30167 Hanover, Germany; 2 NICO: Semanticly, Athens, Greece; 3 Institute for Applied Informatics (InfAI), University of Leipzig, Leipzig, Germany; 4 ZB MED - Information Centre for Life Sciences, Gleueler Straβe 60, 50931 Cologne, Germany

**Keywords:** Research data, Databases

## Abstract

FAIR (meta)data presuppose their successful communication between machines and humans while preserving meaning and reference. The FAIR Guiding Principles lack specificity regarding semantic interoperability. We adopt a linguistic perspective on semantic interoperability and investigate the structures and conventions ensuring reliable communication of textual information, drawing parallels with data structures by understanding both as models. We propose a conceptual model of semantic interoperability, comprising intensional and extensional terminological interoperability, as well as logical and schema propositional interoperability. Since there cannot be a universally accepted best vocabulary and best (meta)data schema, establishing semantic interoperability necessitates the provision of comprehensive sets of intensional and extensional entity mappings and schema crosswalks. In accordance with our conceptual model, we suggest additions to the FAIR Guiding Principles that encompass the requirements for semantic interoperability. Additionally, we argue that attaining FAIRness of (meta)data requires not only their organization into FAIR Digital Objects, but also the establishment of a FAIR ecosystem of FAIR Services, that include a terminology, a schema, and an operations service.

## Introduction

With the total volume of data doubling every three years^1^ and the scientific community experiencing a surge in publications with 7 million academic papers being published annually^2^, it is clear that **we need to harness machine assistance** to avoid that this overwhelming amount of data and knowledge prevents us from gaining meaningful insights and from wasting resources on research that has already been done elsewhere. Currently, a significant portion of research data are still scattered across various repositories and databases, using different data structures and terminologies, challenging researchers not only in discovering and accessing them, but also in integrating them with other data to make them interoperable across different repositories and databases, and finally in reusing them for their specific research interests.

With this in mind, it is essential to facilitate **machine-actionable (meta)data** in scientific research, so that machines can assist researchers in identifying relevant (meta)data pertaining to a specific research question. Moreover, enhancing the machine-actionability of (meta)data could also contribute to a solution to the reproducibility crisis in science^3^ by making raw data available, findable, and reusable^4^.

As a response to the need for machine-actionable (meta)data, in 2016, the **FAIR Guiding Principles for data and metadata** were introduced, providing a framework to assess the extent to which (meta)data are **F**indable, **A**ccessible, **I**nteroperable, and **R**eusable for both machines and humans alike^5^. These principles have gained increasing attention from the research, industry, and knowledge management tool development communities in recent years, and stakeholders in science and research policy have recognized their significance^5–10^. The economic impact of FAIR research (meta)data was estimated by the European Union (EU) in 2018, revealing that the lack of FAIR (meta)data costs the EU economy at least 10.2 billion Euros annually. Taking the positive effects of FAIR (meta)data on data quality and machine-readability into account, an additional 16 billion Euros were estimated^11^. Consequently, the High Level Expert Group of the European Open Science Cloud (EOSC) recommended the establishment of an **Internet of FAIR Data and Services (**IFDS**)**^12^. The IFDS aims to enable data-rich institutions, research projects, and citizen-science initiatives to make their (meta)data accessible in accordance with the FAIR Guiding Principles, while retaining control over ethical, privacy, and legal aspects of their (meta)data (following Barend Mons’ data visiting as opposed to data sharing^13^). Achieving this goal requires the provision of rich machine-actionable (meta)data, the subsequent organization of which into **FAIR Digital Objects (FDOs)**^14,15^, each identifiable by a Globally Unique Persistent and Resolvable Identifier (GUPRI) such as a DOI, Handles, or a resolvable Internationalized Resource Identifier (IRI), and the development of suitable concepts and tools for human-readable interface outputs and search capabilities. While progress has been made in advancing the IFDS, as reflected in efforts by the GO FAIR Initiative, EOSC, and the FAIR Digital Objects Forum, the level of (meta)data FAIRness in many data-intensive institutions and companies is still far from ideal.

The increasing volume, velocity, variety, and complexity of (meta)data pose significant challenges that traditional methods and techniques for handling, processing, analyzing, managing, storing, and retrieving (meta)data are struggling to effectively address within a reasonable timeframe^16^. Knowledge graphs, combined with ontologies and semantic (meta)data schemata, provide a promising technical approach for realizing the FAIR Guiding Principles due to their explicit semantics, structured syntax, and adherence to standardized formats and schemata^17,18^. An **ontology** can be seen as a knowledge base of concepts and their relationships that is built using a formal knowledge representation language that is based on a formal logic such as the Web Ontology Language (OWL) that is based on Description Logics. OWL is the most widely used knowledge representation language for specifying ontologies and has in the meantime become the de facto standard. In the early phase of the history of the Semantic Web research field^19^, ontologies and different versions of Description Logics were developed to provide expressive semantic formalisms for modelling domain knowledge, with the objective to find the right balance between expressivity and reasoning capability. The ontologies had been used primarily for such tasks as discovery, integration, and sharing of (meta)data. In combination with the Resource Description Framework (RDF) as a syntax for specifying directed, labeled, and typed graphs, OWL was used to specify ontologies that then served as schemata in the sense of logics of types for RDF graphs. Many ontologies were developed in this first phase, with the Gene Ontology^20^ or SNOMED CT as prominent examples. Unfortunately, however, most of these ontologies did not live up to their promises, as creating good ontologies consumed substantial resources, their re-usability was limited, and their effect on the overall interoperability of (meta)data across the Web was smaller than expected, resulting only in small islands of interoperability instead of an overall interoperable Web.

**Knowledge graphs** came up in the early 2010s, following the Linked Open Data (LOD) phase of the history of the Semantic Web^19^ that focused on developing technologies for accessing and querying data and knowledge over the Internet and that predominantly used more simplistic ontologies (e.g., schema.org that is widely used for annotating Web pages) for providing only basic schemata for structuring RDF graphs. The term knowledge graph first emerged in 2012, following the launch of Google’s knowledge graph and the subsequent development of similar knowledge graphs by other companies. These industry knowledge graphs employ ontologies and LOD technology but lack the openness of LOD graphs such as Wikidata^19^. In contrast to the ontologies from the initial phase, which adopted a top-down approach for the structuring of (meta)data and knowledge, the industry knowledge graphs are distinguished by a bottom-up approach to the specification of **semantic (meta)data schemata**. On the other hand, they are not created following an open, community-driven approach, such as Wikidata, but instead, can be characterized by their **central control**. Consequently, in contrast to LOD graphs, these knowledge graphs are more integrated and internally more consistent, with a strong focus on proper modelling using adequate ontologies and semantic schemata. As industry artefacts, these knowledge graphs are designed to serve a specific use case and support particular tasks, often requiring (meta)data in the graph to be highly **interoperable** and readily **machine-actionable** with respect to these tasks, having ontologies combined with semantic (meta)data schemata as the most recurrent solutions for semantic interoperability issues^21^. For a more detailed description of the history of the Semantic Web, see^19^.

Knowledge graphs represent instances, classes, and their relationships as resources with their own GUPRIs. These GUPRIs are used to denote relationships between entities using the triple syntax of *Subject*-*Predicate-Object*. Each relationship is thus modeled as a structured set of three distinct elements (i.e., data points), taking the form of resources and literals. Class resources for types of entities and schemata for modelling their universal relationships are provided by ontologies, whereas schemata for modelling assertional and contingent relationships between individual entities are provided by additional semantic (meta)data schemata. Knowledge graphs are stored in graph databases. In contrast, in relational databases, entity relationships are modelled between data table columns and not between individual data points. Consequently, the graph databases of knowledge graphs outperform relational databases in handling complex queries on densely connected data^22^, which is often the case with research data (with the exception of highly dimensional numerical data requiring complex analytical operations where knowledge graphs may face scalability challenges^23^). Moreover, relational databases aim to record information that constitutes a datum (e.g. a particular empirical measurement), while the graph databases of knowledge graphs, additionally, also typically represent the relationships of all entities referenced in a datum, ideally reflecting the relationships of the corresponding real-world entities and thus representing a kind of digital twin. Although knowledge graphs are not the only solution for achieving FAIR (meta)data, and although the popularity and success of the FAIR Guiding Principles is at least partly due to the fact that they are formulated independently of any specific technological solutions, the graph databases of knowledge graphs are due to these characteristics particularly well suited for FAIR research and development and for all tasks requiring efficient and detailed retrieval of (meta)data.

Nonetheless, employing ontologies and knowledge graphs to document (meta)data does not in itself guarantee adherence to the FAIR Principles. Achieving FAIRness necessitates meeting additional requirements, such as consistent usage of the same semantic (meta)data schema for identical types of (meta)data statements to ensure their schema interoperability^24^, as well as organizing (meta)data into FAIR Digital Objects^14,15^. Moreover, knowledge graphs, being a relatively new concept and technology, introduce their own specific technical, conceptual, and societal challenges. This is evident in the somewhat ambiguous nature of the knowledge graph concept^17^ and the lack of commonly accepted standards, given the diverse technical and conceptual incarnations ranging from labeled property graphs like Neo4J to approaches based on RDF, employing RDF triple stores and applications of Description Logics using OWL (but see Hogan *et al*.^25^ for a convincing definition of knowledge graphs).

In this paper, we use FAIR knowledge graphs to refer to knowledge graphs as machine-actionable semantic graph structures designed to document, organize, integrate, and represent different types of information, including lexical, assertional, contingent, prototypical and universal statements. And we distinguish them from ontologies, which focus primarily on universal and lexical statements without incorporating assertional and contingent statements. **Lexical statements** (*terminological statements* sensu^26^) are about linguistic items such as terms in a controlled vocabulary and comprise information such as the label, identifier, the human-readable definition of a term, and specifications of synonyms. In ontologies, lexical statements are usually documented using annotation properties. **Assertional statements** state what is the case (e.g., *This swan is white*). They are statements that are true for specific particulars. Empirical data are assertional statements. **Contingent statements** state what can be the case (e.g., *Swans can be white*). They are only true for some instances of a specific type of entity. **Prototypical statements** are a subcategory of contingent statements and state what is typically the case (e.g., *Swans are typically white*). They are considered to be true as long as not the contrary is explicitly stated. **Universal statements** state what is necessarily the case (e.g., *All swans are white*). They are true for every instance of a specific type of entity.

Ontologies, in turn, can vary considerably in quality. Their correct logical application is not straightforward and requires expertise in semantics. Consequently, there is a need for tools that quantitatively and transparently measure the FAIRness of research (meta)data. In this context, it is important to note that the **FAIR Guiding Principles are not a (meta)data standard** themselves, but a list of criteria that must be followed to obtain FAIR (meta)data—they do not specify, how FAIRness must be achieved. However, the popularity of the Principles triggered and informed the development of various FAIRness assessment tools that now start to serve as practical benchmarks within different research communities. Especially **automated FAIRness assessment tools** such as FAIR-Checker^27^, F-UJI^28,29^, and FAIR Evaluator^30^ are of particular importance as they provide transparent workflows for evaluating FAIRness scores and are likely contributing to the establishment of corresponding new (meta)data standards. These automatic tools have in common that they are limited to assessing only the FAIRness of basic provenance (e.g., creator, creation date) and licensing metadata (i.e., copyright license), but do not assess the FAIRness of domain-specific (meta)data. This is understandable, as they assess the FAIRness against a defined set of relevant and well-established vocabularies, which are easier to specify for this kind of metadata but not as straightforward for domain-specific (meta)data if the assessment tool should be a general and not a domain-specific solution. However, the use of these automated FAIRness assessment tools is insofar problematic as they can give the impression to provide a general FAIRness score for a given dataset for which only the FAIRness of this basic metadata has been assessed. Since the FAIR Guiding Principles explicitly state that they apply to both data and metadata of any kind, such assessments would be misleading.

A note regarding the terminological conventions we use in this paper: “triple” refers to an RDF triple statement consisting of subject, predicate, and object, while “statement” refers to a natural language statement. Also, when we talk about “schemata”, we explicitly include schemata for statements and for collections of statements and not only schemata for individual triples. Furthermore, for ease of understanding, all resources in the text and figures are represented by human-readable labels rather than their GUPRIs. It is implicitly assumed that each property, instance, and class is uniquely identifiable by a GUPRI.

## Machine-Actionability

The concepts of machine-actionability and interoperability of (meta)data are central to this paper. We therefore want to clarify what we mean by them. Weiland *et al*.^31^ gave the following definitions:

**Machine-readable** “*are those elements in bit-sequences that are clearly defined by structural specifications*” (p. 3), such as data formats like CSV, JSON, or XML, or resources (i.e., IRIs) and literals with datatype-specifications as they are used in the Resource Description Framework (RDF).

**Machine-interpretable** “*are those elements* [in bit-sequences] *that are* ***machine-readable*** *and can be related with semantic artefacts in a given context and therefore have a defined purpose*” (p. 3, emphases added), such as referencing defined and registered ontology terms that provide meaning to a resource.

**Machine-actionable** “*are those elements in bit-sequences that are* ***machine-interpretable*** *and belong to a type* [of element] *for which* ***operations*** *have been specified in symbolic grammar*” (p. 3, emphases added), thus linking types of (meta)data statements to operations such as logical reasoning based on Description Logics for OWL-based (meta)data and other rule-based operations such as unit conversion or other (meta)data analyses.

Looking at these definitions, it is clear that **machine-actionability cannot be simplified as a mere Boolean property**. Instead, it exists on a spectrum, allowing for **degrees of machine-actionability**. Numerous operations can potentially be applied to a given set of (meta)data, and the ability to apply even a single operation would otherwise suffice to classify the (meta)data as machine-actionable. Additionally, machine-actionability does not describe an intrinsic quality of (meta)data but rather a disposition to take a specific role in a specific operation that can be executed by a machine. As machine-actionability depends on operations, we understand machine-actionability as a particular relationship between (meta)data and data ecosystems. Consequently, specifying the set of operations that can be applied to the (meta)data, along with the corresponding tools or code (i.e., *dataset A is machine-actionable with respect to operation B using tool C*), is more meaningful than simply labeling it as machine-actionable.

Achieving machine-actionability also entails the capacity to identify the specific type of (meta)data within a given dataset that is amenable to processing through a particular operation conducted by a specific tool or application, and vice versa. For example, identifying all subgraphs in a FAIR knowledge graph or all columns in a table in a relational database that model measurement data to which a particular statistical test operation can be applied.

It is worth noting that a machine reading a dataset could be regarded as an operation in itself. Datasets documented in formats such as PDF, XML, or even ASCII files could be considered machine-readable and, to some extent, therefore also machine-actionable. Moreover, if a dataset is machine-readable, search operations can be performed on it as well, enabling the identification of specific elements through string matching, for example. The success of search operations serves as a measure of the findability of (meta)data. Machine-readable (meta)data can be found through string matching, while interpretable (meta)data can be found through their meaning, referent, or contextual information. Thus, (meta)data that are readable but not interpretable possess limited findability. Consequently, analog to machine-actionability, **findability cannot be characterized as a Boolean property but rather manifests in degrees**.

It is important to emphasize that the definition of machine-actionability we refer to, as outlined above, strictly depends on machine-interpretability. Consequently, machine-reading of a dataset and machine-searching based on string matching are not considered proper examples for operations that fulfill the requirements of machine-actionability.

Guizzardi^32^ argues that (meta)data invariably entails ontological commitments, and in instances where these commitments are under-constrained, the resultant datasets can provide semantically equivalent representations of reality, thereby giving the erroneous impression of ontological equivalence, despite the divergent intentions of their respective creators. This phenomenon occurs when for instance the same term is used across multiple datasets with differing intended meanings. Guizzardi therefore underscores the significance of ontologies, particularly **foundational ontologies**, in serving as formal, shared, and explicit representations of fundamental conceptualizations for the documentation of information. Foundational ontologies offer domain-independent formal theories that provide representations of general aspects of reality, like for instance identity, dependence, parthood, truthmaking, and causality. These aspects provide a conceptual foundation for the development of domain-specific conceptualizations. Formal ontology languages that are used to describe these foundational ontologies are grounded in a formal logical framework, such as First Order Logic or Description Logics. Guizzardi’s argument asserts that semantic interoperability for (meta)data, and consequently machine-actionability, can only be achieved through the utilization of ontologies that are grounded in foundational ontologies for their representation^32^.

Therefore, machine-interpretability of (meta)data is contingent on ontologically consistent conceptualizations. This entails the requirement that (meta)data structures must explicitly disclose their underlying ontological commitments. Given that machine-actionability of (meta)data is dependent on their machine-interpretability, it is also dependent on their semantic interoperability. The utilization of sound domain ontologies that are grounded in robust foundational ontologies for documenting (meta)data offers a promising solution for attaining their semantic interoperability.

## Interoperability

Interoperability has been an important research topic for at least the last four decades. Interoperability of (meta)data, as we understand it, is closely related to machine-actionability in the sense that datasets *A* and *B* are considered *C*-interoperable if a common operation *C* exists that can be applied to both. However, contrary to machine-actionability, interoperability does not require that a machine can conduct this operation as it can also be conducted by a human.

Interoperability is thus more general than machine-actionability, and machine-actionability is a specific type of interoperability. Interoperability of (meta)data passes on to machine-actionability that it is not describing an intrinsic quality of (meta)data but a disposition to take a specific role in a specific operation. The interoperability of (meta)data therefore describes a particular relationship between (meta)data and data ecosystems and between (meta)data and their user communities. This characterization goes beyond the more generic definition of interoperability as the capability of two or more systems or datasets to exchange and use information for a specific purpose^33^, including their ability to exchange information without creating technological dependencies between them^34,35^, as it includes the perspective of humans as potential users of (meta)data. We thus understand the set of all machine-actionable datasets as a subset of all interoperable datasets, and the set of all interoperable datasets as the combination of all machine-actionable and all human-actionable datasets.

To achieve interoperability in the context of the exchange of (meta)data between different agents (i.e., machines and/or humans), (meta)data must be transferred in a way that guarantees that they remain functional and processable in the receiving agents, which, in turn, requires (meta)data service standardization^36^. Interoperability also passes on to machine-actionability that it is not a Boolean property, but rather exists along a continuum, determined by the range of different types of operations that can be executed on a given type of (meta)data.

(Meta)data are composed of terms that form statements. With terms, we here refer to strings of literals and symbols that identify or represent real-world kinds, concepts, individuals, or values. The weight measurement of an apple *A*, for instance, is composed of terms representing the individual apple *A*, its particular weight, the measured value and the measurement unit, forming the statement *‘Apple A has a weight of 212.45 grams’*. Both terms and statements play a crucial role in conveying semantic content and thus meaning, forming the basis for successfully communicating information. Consequently, the interoperability of terms and of statements between sender and receiver of information is essential for effective communication. Exchanging (meta)data between machines, between machines and humans, and between humans is not only about guaranteeing their readability but also involves processing to ensure their interpretability, i.e., receiver understands their intensional meaning, and their actionability.

Evidently, interoperability plays a crucial role in facilitating this communication process and is central to the realization of FAIR (meta)data. Without interoperability, the findability and reusability of (meta)data are limited, and without interoperability there is no machine-actionability. The significance of interoperability has also been duly acknowledged by the EOSC. In their **EOSC Interoperability Framework**^14^, which draws inspiration from the European Interoperability Framework developed by the European Commission in 2017, they delineate, in accordance with the FAIR Guiding Principles, four distinct types of interoperability (they call them *layers*) for scientific data management:

- **technical interoperability** oversees interoperation at the application level within an infrastructure (i.e., information technology systems must work with other information technology systems in implementation or access without any restrictions or with controlled access);
- **semantic interoperability** is concerned with achieving shared semantic understanding of (meta)data elements (i.e., contextual semantics related to common semantic resources);
- **organizational interoperability** focuses on harmonizing business processes (i.e., contextual processes related to common process resources); and
- **legal interoperability** guarantees cooperation among organizations operating under diverse legal frameworks, policies, and strategies (i.e., contextual licenses related to common license resources).

Alternative differentiations of interoperability have been suggested. Sadeghi *et al*.^36^, for instance, distinguish the following three types of interoperability (they call them *facets*) in their *interoperability trilogy*, in relation to needs they identified based on an interoperability survey they conducted:

- **data interoperability**: the need to exchange (meta)data, which is challenged by heterogeneous (meta)data;
- **service interoperability**: the need to use one another’s services, which is challenged by disparate APIs and services;
- **system interoperability**: the need to systematically manage the infrastructures for distributing, discovering, sharing, and exchanging artefacts, which is challenged by historical constraints and the lack of common practices to develop modular systems and the lack of tools supporting modular software engineering.

Interoperability covers a wide range of different dimensions and is increasingly understood as a multidimensional concept. Many interoperability frameworks with different types of interoperability have been suggested. In their comprehensive tertiary study, Maciel *et al*.^21^ identified 36 different types of interoperability and organized them into these three groups:

- **technological**: types of interoperability that address interactions among elements of software-intensive systems (e.g., hardware, networks, software platforms), with the purpose of resolving heterogeneity *within* software infrastructures (e.g., technical, objects, and device-level considerations) or facilitating collaboration *between* software elements, addressing semantic, syntactic, and pragmatic requirements, including
  - **semantic interoperability**: requires systems to agree on a shared standard of representing information to enable them to efficiently communicate information without human intervention;
  - **syntactic interoperability**: refers to how information is exchanged, requiring standardized structures and protocols;
  - **pragmatic interoperability**: refers to the aims of communicating information by transparently specifying context, intention, and effect of information exchange;
- **social-technical**: types of interoperability that enhance the ability of entities external to (meta)data structures or software-intensive systems such as people and organizations to collaborate effectively, resolving their differences, including
  - **individual interoperability**: challenges relating to human interactions and their cultural difference;
  - **organizational interoperability**: challenges relating to differences in governance and internal company processes;
- **crosscutting**: types of interoperability that influence both technological and social-technological dimensions, with **legal interoperability** as the only example.

According to Maciel *et al*.^21^, most interoperability frameworks focus on technical, syntactic, and semantic interoperability as the most important types of interoperability for developing interoperable software systems. In what follows, we focus on **semantic interoperability** and the facet of **data interoperability** and compare it to conditions required for semantic interoperability between humans communicating with each other using natural language.

## Outline

Whereas many discussions around the practical implementation of the FAIR Guiding Principles focus on the FAIRness of basic provenance and licensing metadata, our main focus in this paper lies on the question of what the requirements are for establishing **FAIRness** not only across all kinds of metadata but also **across all kinds of data**.

The starting point of our considerations is the supposition that the **original FAIR Guiding Principles were conceptualized in the spirit of LOD**, with a strong focus on the use of interlinked GUPRIs (i.e., IRIs), FAIR vocabularies (i.e., simplistic ontologies and other controlled vocabularies), and a formal, shared, and broadly applicable knowledge representation language (e.g., RDF) for documenting (meta)data (cf.^5^). We postulate that this results in the same challenges encountered by LOD regarding general reusability and the integration of (meta)data, thereby impeding the realization of the underlying objective of an IFDS characterized by shared, discoverable (meta)data that are integrated and reusable. The shift in focus from LOD to (industry) knowledge graphs that has taken place in the third phase in the history of the Semantic Web research field has been driven by practical needs for (meta)data to be more interoperable and readily machine-actionable and these needs should be reflected in the FAIR criteria.

We assume that the demand for FAIR and machine-actionable (meta)data presupposes their **successful communication between machines and between humans and machines**, which ultimately always boils down to **meaning-preserving communication of information between humans**, whether mediated by machines and information structures or not. During such communication processes, preserving the meaning and reference of the message between sender and receiver is crucial, requiring both parties to share the same background knowledge, encompassing lexical competencies, syntax and grammar rules, and relevant contextual knowledge. Based on this assumption, we analyze the different aspects that affect the machine-actionability and semantic interoperability of (meta)data and identify criteria for semantic interoperability that facilitate discovering, sharing, integrating, and reusing (meta)data. We also shed some light on why achieving semantic interoperability across data management systems is such a big challenge. Part of the reason is that semantic interoperability requirements go beyond simply mapping terms across different controlled vocabularies. We also must consider what is required for establishing semantic interoperability of statements and sets of statements, and thus granularity levels of information coarser than the level of individual terms.

In the *Result* section, we first draw inspiration from natural languages like English and explore how semantic interoperability can be understood by going into some detail in analyzing the way terms and natural language statements convey meaning and information, providing a **linguistic perspective on semantic interoperability**. Recognizing terms and statements as basic units of information, we investigate the **linguistic structures** that ensure reliable communication of information and draw parallels with the structures found in data schemata as the machine-actionable counterparts to statements, understanding both as **models** that model some referent system. When reading this section, you may ask yourself how this relates to (meta)data, FAIRness, semantic interoperability, and machine-actionability, but we believe that this analysis yields insights that help us to better understand the complexity of semantic interoperability and what is required for achieving it for terms and statements and provides an important perspective on semantic interoperability in general that must be taken into account in any interoperability framework. As a result of this analysis, we outline a **conceptual model of semantic interoperability** that distinguishes terminological and propositional interoperability as the two main aspects of semantic interoperability. Terminological interoperability involves sharing the same intensional and extensional meaning, propositional interoperability involves sharing the same data schema and logical formalism.

Whereas many discussions around the practical implementation of the FAIR Guiding Principles were centered on the FAIRness of basic provenance and licensing metadata, our main focus in this paper lies on investigating the requirements for establishing FAIRness across a diverse array of data. We argue that there is no simple solution for establishing cross-domain FAIRness of (meta)data. It is neither possible to develop a single best ontology for all domains of research and for the various purposes researchers are interested in and the different scientific frames of reference they adopt, nor is it possible to develop a set of best data schemata for all kinds of operations one wants to conduct on (meta)data. Thus, semantic interoperability issues will inevitably remain to exist in the future. We therefore **suggest additions to the FAIR Guiding Principles towards FAIR 2.0**, which reflect, among other things, requirements on (meta)data that we identified in our conceptual model of semantic interoperability. Because FAIR knowledge graphs combined with ontologies and semantic schemata for (meta)data offer themselves as a promising solution for the problems inherent in LOD, we use them as an example throughout this paper. To keep true to the spirit of the original FAIR Guiding Principles, however, the suggestions for the additions to FAIR are phrased in a manner that is as technology-independent as possible.

In the *Discussion* section, we argue that for achieving FAIRness of (meta)data, we need **FAIR Services** in addition to organizing data in FDOs, since the interoperability and FAIRness of (meta)data not only depends on the availability of readily applicable operations (i.e., machine-actionability), but also on the provision of a comprehensive set of relevant intensional and extensional entity mappings and schema crosswalks. We discuss that the FAIR Services therefore must include a terminology service, a schema service, and an operations service.

## Results

### Semantic interoperability and what natural languages like English can teach us

The EOSC Interoperability Framework characterizes semantic interoperability as a requirement for enabling machine-actionability between information systems, and it is achieved *“when the information transferred has, in its communicated form, all of the meaning required for the receiving system to interpret it correctly”* (p. 11). According to Maciel *et al*.^21^, semantic interoperability “*provides communication without information ambiguity and in an accurate way without human intervention, so software systems need to agree with a common information representation*” (p. 12). Guizzardi^32^ characterizes semantic interoperability as “*a relation between worldviews, or more technically, a relation between* Conceptualizations” (p. 182, emphasis from the original); “*two systems A and B semantically interoperate if the coded relations connecting the information structures of A and B: (i) preserve the semantics of the referents represented in those structures; (ii) reflect the real-world meta-properties of the represented relations; and (iii) yield a resulting information structure that constraints the possible states of the resulting system to the intended one, i.e., to those that represent intended state of affairs according to the conceptualizations underlying A and B*” (p. 185).

All of these characterizations have in common that they focus on the machine-actionability aspect of semantic interoperability. As previously outlined, the interoperability of (meta)data is predicated on successful communication between machines and between humans and machines, as well as between humans. In this section, we endeavor to illuminate the human-actionability aspect of semantic interoperability. To elucidate the conceptual underpinnings of semantic interoperability, it is instructive to examine how humans communicate meaning (i.e., semantic content) in a natural language such as English, employing terms and statements as the basic meaning carrying units of information. It is imperative to acknowledge the limitations of our argument by emphasizing English as a specific example of a natural language. We recognize the substantial variations in syntax across different natural languages and do not assert the universal validity of our argument for all natural languages. With the term “communication”, we mean the attempt to establish a congruent cognitive representation of information between the sender and receiver, employing textual representational artifacts such as terms and statements, as mediators.

#### Requirements for successfully communicating terms and statements

In order to facilitate effective and reliable communication it is essential to understand the elements that enable successful interaction between individuals. A prerequisite for successful communication is the presence of a shared background of relevant knowledge among the sender and the receiver. This necessitates an examination of the characteristics that define the readability, interpretability, and actionability of natural language terms, prior to the analysis of natural language statements.

##### Interoperability of terms

We can say that a **term** as a textual representational artifact (i.e., a linguistic entity) is **readable** if it consists of a sequence of characters that can be assigned to sounds (for the sake of simplicity, we will limit ourselves to terms and no other symbols, such as emojis). Following this notion, ‘*EGrzjEZhsmtrjE*’ would be a readable term for most English readers, although the term would likely be meaningless to them, whereas ‘*Це можна прочитати*’ is not readable, because it uses the Cyrillic alphabet which most English readers are not familiar with. Following this notion of readability, sender and receiver must use the **same set of characters** (i.e., the same alphabet) and agree on an **order** and thus a **reading direction** for communicating terms.

**Fig. 1:**
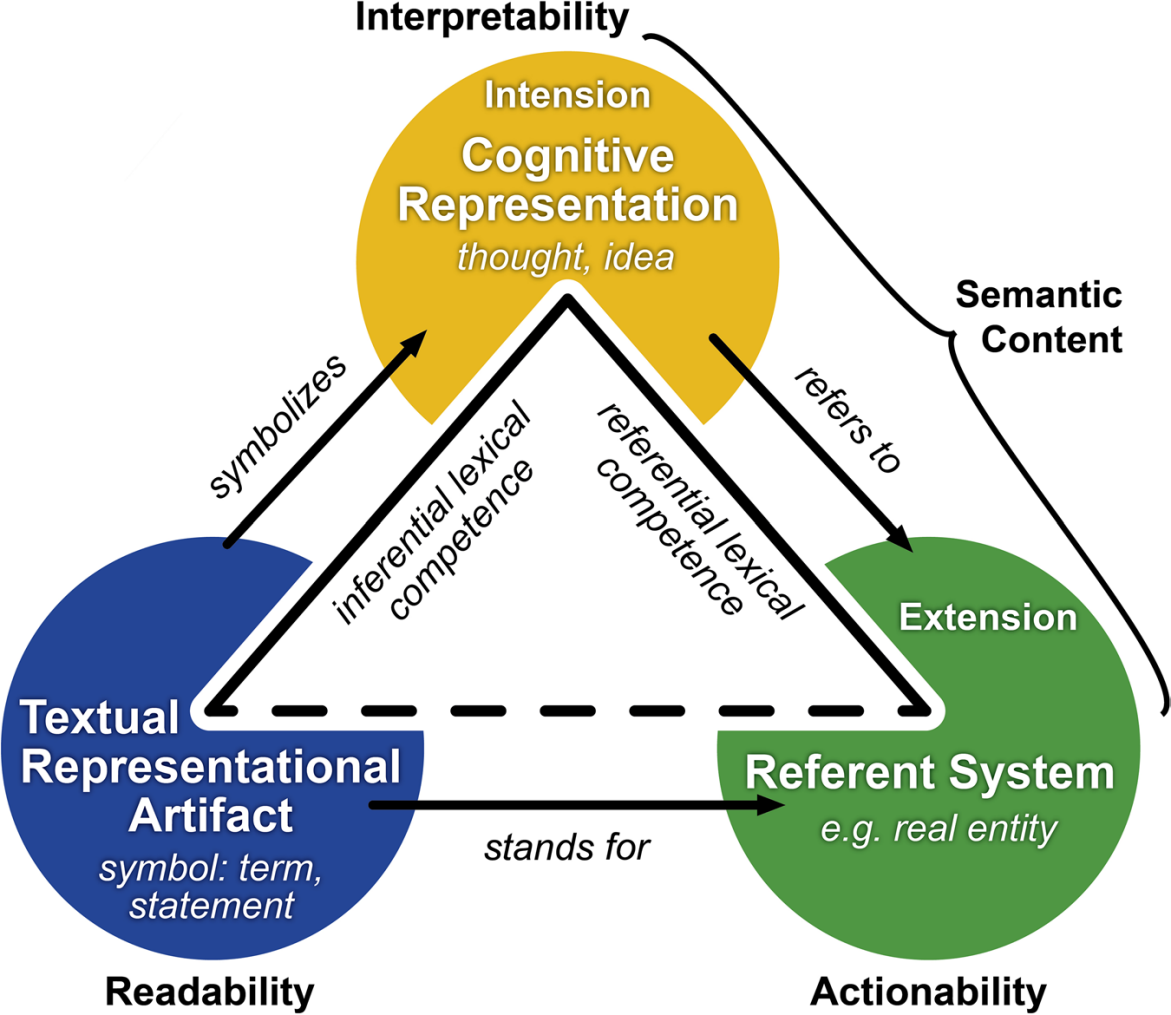
Semiotic triangle. The semiotic triangle displays the relations between a textual representational artifact^72,73^ in the form of a linguistic symbol, such as a specific term or statement, its corresponding cognitive representation in the form of a thought or idea that provides the intensional meaning for the symbol (based on Frege’s *sense*), and its corresponding referent system that provides the extensional meaning for the symbol (based on the term’s reference). The textual representational artifact does not directly relate to its corresponding referent system but is always mediated by a corresponding cognitive representation. The combination of the corresponding intensional and extensional meaning represents the semantic content (i.e., *meaning* in a general sense) of a textual representational artifact. With inferential and referential lexical competence, the two requirements for successfully communicating the semantic content of a term are specified. We adapted the diagram from^74^, incorporated the distinction between textual representational artifact, cognitive representation, and (real) referent system from^72,73^, and modified it to correspond with our use case. The original semiotic triangle follows Peirce’s^75^ and Frege’s^39,40^ independently developed triadic notion of a sign.

The readability of a term can be contrasted with its interpretability. We call a term as a textual representational artifact **interpretable**, if it consists of a sequence of characters that are **readable** and that can be assigned to a **corresponding cognitive representation** (see Fig. 1). Following this notion, ‘*EGrzjEZhsmtrjE*’ is readable but not interpretable, whereas the term ‘*tree*’ is both readable and interpretable for most English readers. Typical examples of readable but not universally interpretable terms are acronyms such as ‘*RDF*’. Therefore, the intensional meaning of an acronym is usually introduced before the acronym is used in communication. Interpretability of a term is achieved if sender and receiver share the same **inferential lexical competence**^37^ in the form of knowledge about the **intensional meaning** of the term, so that interpreting the term generates the same cognitive representation in the receiver as intended by the sender. Inferential lexical competence refers to the ability to understand and make inferences about the intensional meaning of words and phrases based on contextual clues and linguistic knowledge. **Ontological definitions** that answer the question ‘*What is it?*’^38^ and **intensional definitions** that answer the question ‘*How is it understood, presented, or interpreted?*’ provide such knowledge, with the latter referring to Frege’s^39,40^ understanding of *sense* that is based on the way a term or expression presents a concept or object and thus the mode of representation or the perspective under which the reference is understood. Such as the terms ‘Morning Star’ and ‘Evening Star’, which have the same referent (planet Venus) but different senses because they describe Venus in different ways.

A term as a textual representational artifact is **actionable**, if it consists of a sequence of characters that is **interpretable** and to which the operations ‘**designation**’ (i.e., the object is given, and the matching term must be found) and ‘**recognition**’ (i.e., the term is given, and the matching object must be identified) can be applied. These operations require the sender and receiver to share the same **referential lexical competencies**^37^ and thus **diagnostic knowledge** about the referent/extension of a given term. The referent/extension of a term is thus the referent system (e.g., a real entity) to which the term refers (see Fig. 1). A proper name refers to an individual entity (e.g., the referent of the term *Earth* is the planet Earth) and a general term or kind term to a set of individuals, i.e., the instances of that term (e.g., the extension of the term *planet* are all planets that have existed and will exist). Referential lexical competence is the ability of a language user to understand and use words in a manner that accurately refers to objects, concepts, or phenomena in the world.

**Diagnostic knowledge** is often communicated in the form of **method-dependent recognition criteria**, images, or by referring to exemplars, and it answers the question ‘*How does it look, how to recognize or identify it?*’^38^, enabling the receiver to use the term correctly in designation or recognition tasks. Unfortunately, many ontologies only provide ontological/intensional definitions and no method-dependent recognition criteria. For example, the term ‘*cell nucleus*’ (FMA:63840) of the Foundational Model of Anatomy ontology^41^ is defined as “*Organelle which has as its direct parts a nuclear membrane and nuclear matrix*”. As we cannot see cell nuclei without staining a cell sample and using a microscope, the definition does not provide the practical diagnostic knowledge needed for designation and recognition tasks. The term therefore does not provide the information needed to build the referential lexical competencies required to make the term human-actionable. Referential lexical competencies are thus needed to use a term correctly in different contexts. They are essential for the **human-actionability of terms**.

The difference between inferential and referential lexical competence can be nicely illustrated by transferring these two concepts to the daily work of physicians: In many cases, a physician’s ontological knowledge (c.f., inferential lexical competence) of bacteria in general is sufficient to know that a bacterial infection can likely be fought successfully with broad-spectrum antibiotics. However, having only general diagnostic knowledge of bacteria is not sufficient to reliably recognize (c.f., referential lexical competence) Lyme disease, for example, since symptoms of bacterial infections are often bacterium- and host-specific and can vary substantially across different bacteria.

##### Interoperability of statements

Given that the intensional meaning of a term is provided by its ontological and/or intensional definition, which in turn takes the form of one or more statements, one could argue that terms are only placeholders, i.e., surrogates, for their definitions and thus for statements, and that only statements carry meaning. While this may be a stretch, the communication of meaning requires more than just terms, but also rules and structures to place several terms in a specific meaningful context, related by predicates.

**Statements are endowed with meaning in addition to the meaning of the terms that compose them**. This phenomenon becomes evident when the same set of terms is rearranged, resulting in divergent meanings, as illustrated by the sentences *“Peter travels from Berlin to* *Paris”* and *“Peter travels from* *Paris* *to Berlin”*. Consequently, for the efficient and reliable communication of statements, the sender and the receiver of the information must adhere to a set of rules and conventions for sentence formulation using terms.

But how is the intensional meaning of a statement represented in the human brain? Understanding how the human brain creates cognitive representations of semantic content is an active area of research^42^. The human brain is a highly interconnected complex system that is continually influenced by input signals from the body and the world, so that a given neuron does not function in isolation but is substantially influenced by its neural context^43^. Evidence suggests that the human brain represents object knowledge through at least two distinct systems: one based on motor and sensory experiences, and the other rooted in conceptual and cognitive processes^44,45^. It is therefore reasonable to assume that lexical concepts are stored as patterns of neural activity that are encoded in multiple areas of the brain, including taxonomic and distributional structures as well as experience-based representational structures that encode information about sensory-motor, affective, and other features of phenomenal experience^46^. These findings suggest that the cognitive representation of the intensional meaning of a statement is likely to take the form of a complex network of associations, analogous to a multidimensional mind-map. Thus, when attempting to communicate a statement, the sender must first translate this multidimensional mind-map into a one-dimensional sequence of terms, i.e., a sentence, and the receiver must then translate it back into a multidimensional mind-map. These two translation steps are supported by a set of syntactic and grammatical conventions, shared by the sender and the receiver, for formulating sentences using terms.

According to the **predicate-argument-structure** of linguistics^47,48^, the main verb of a statement, together with its auxiliaries, forms the statement’s predicate. A predicate has a **valence** that determines the number and types of subject and objects, called **arguments** (not to be confused with arguments in the sense of debates), that it requires to complete its meaning. Further objects, called **adjuncts**, may be additionally related to the predicate, but they are not necessary to complete the predicate’s meaning. In this context, an argument is a subject or an object that is required for a given predicate to form a semantically meaningful statement, whereas adjuncts can be removed from a statement and the statement is still meaningful. Adjuncts thus provide optional information, such as a time specification in a material parthood statement—you can remove the time specification (adjunct) from a material parthood statement and the statement still makes sense, whereas removing the material object that designates the part (argument) would result in a nonsense parthood statement. Every statement has a subject phrase as one of its arguments and can have one or more object phrases as further arguments and additional object phrases as adjuncts, depending on the underlying predicate.

**Fig. 2:**
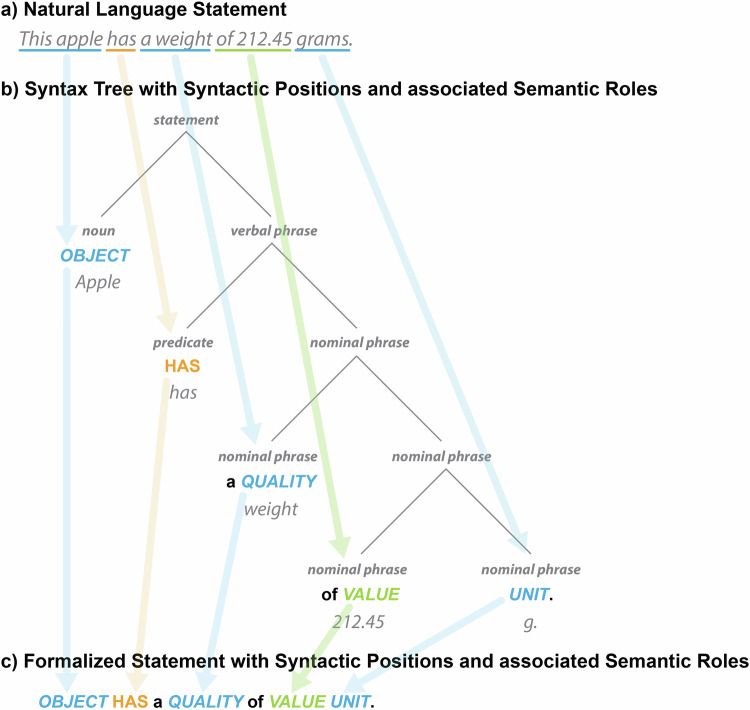
Structure underlying a natural language statement. The natural language statement in (**a)** is structured by syntactic and grammatical conventions into syntactic positions of phrases of a syntax tree as shown in (**b)** or a formalized statement as is shown in (**c)**, where each position possesses a specific associated semantic role that can be described by a thematic label.

Each argument and adjunct of a predicate can be understood as having a specific **syntactic position** within the statement (i.e., position in a syntax tree), with each position having its own specific **semantic role** that the position expresses (Fig. 2b; see also Kipper *et al*.’s^49^ *thematic roles* and their verb lexicon VerbNet, which extends Levin verb classes^50^). The list of subject and object arguments of a predicate-argument-structure can be described by a list of **thematic labels**, each reflecting the semantic role of its syntactic position (e.g., OBJECT, QUALITY, VALUE, UNIT in Fig. 2), and the syntactic structure of a statement can be represented by an ordered sequence of such thematic labels (Fig. 2c). The thematic labels thus function as descriptors of semantic roles that are mapped onto positions in a given syntactic frame^49^ (see also PropBank^51^).

Syntax trees, with their different syntactic positions and associated semantic roles, contribute substantially to the meaning of their sentences, and they are used to translate a cognitive representation in the form of a web of ideas in the mind of a sender into a textual representational artifact in the form of a string of words that can be understood by a receiver, who translates them back into a cognitive representation in the form of a web of ideas^52^. The clearer the semantic roles of the different positions are, the easier it is for humans to understand the information.

In a sense, when considering that different syntax trees can share the same thematic label, which then interconnects them (i.e., the object of one sentence is the subject of another), we can understand graphs of interconnected syntax trees as the first knowledge graphs created by humans, and their use seems to be quite straightforward, providing a structure that is interoperable with human cognitive conditions.

With all this in mind, we can now state that a **statement** as a textual representational artifact is **readable** if it consists of a sequence of characters that can be assigned to terms and sounds, with **rules** delineating the **termination of a term** and the **conclusion of a statement**. The statement ‘*EGrzjEZ hsmtrjE*.’ would be deemed readable, with the space and the period serving as markers for the termination of a term and the statement’s conclusion, respectively. Conversely, ‘*EGrzjEZhsmtrjE*’ is not a readable statement due to its lack of this essential information, and would be read as a single term rather than a statement comprising of several terms.

A statement is **interpretable**, if it consists of a **readable sequence of terms**, each of which is **interpretable** and ideally also **actionable** and that, based on **conventions**, can be assigned to a **syntax tree** with **syntactic positions** and **semantic roles** and thus to a particular intensional meaning. The intensional meaning of a statement, however, goes beyond the intensional meaning of the set of its terms. Someone can read “*The Waste Land*” from T.S. Eliot and understand every single word of it but can still not understand its intensional meaning due to its fragmented structure and references to multiple literary and cultural traditions. However, as a minimum requirement for the interoperability of a statement, sender and receiver must agree on a **common statement structure** with positions for terms and a **shared terminology**.

And finally, a statement is **actionable**, if it consists of a sequence of terms that can be **interpreted as a statement** and to which the operations of ‘**recognition**’ (i.e., the statement is given and the matching situation must be identified) and ‘**description**’ (i.e., a situation is given, and the matching statement describing it must be found) can be applied. Moreover, actionable statements can potentially be formed together with other statements into a **meaningful narrative**. This requires sender and receiver to agree on rules on how to refer to entities and semantic content already mentioned in previous statements, using for example pronouns such as ‘*they*’ and ‘*this*’.

**Fig. 3:**
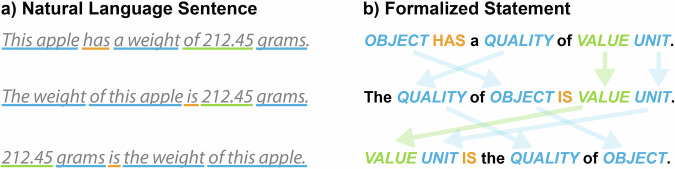
Different expressions of the same proposition. (**a)** Three different expressions (i.e., natural language sentences) of the same proposition, all identical in their intensional meaning. (**b)** The formalized statement for each statement from the left, with each syntactic position represented by its associated thematic role. The alignment of positions that share the same thematic role across the three statements is indicated by arrows.

Given the high degree of **expressiveness** characteristic of natural languages, any given proposition (i.e., statement) can typically be expressed in multiple sentences. As demonstrated in the above example, the sentence *‘This apple has a weight of 212.45 grams’* (Fig. 2) can be expressed in a variety of ways, including *‘The weight of this apple is 212.45 grams’* or *‘212.45 grams is the weight of this apple’*. Despite these three sentences having significantly different structures, by intuitively mapping the semantic roles associated with different syntactic positions across these sentences and by identifying that each position associated with the same role shares the same content, all three sentences can be recognized immediately as communicating the same intensional meaning (see Fig. 3). Therefore, despite the utilization of varied expressions to convey the same information, semantic interoperability is not typically compromised.

In essence, effective and reliable communication of information necessitates the ability of the sender and receiver to identify terms and statements as the fundamental units of information in a message, recognizing their initiation and termination for optimal **readability**. Furthermore, it is imperative that both parties possess analogous inferential lexical competencies concerning the terms employed in their communication, along with shared syntactic and grammatical conventions for sentence formulation. This ensures the generation of equivalent syntax trees in both parties, thereby facilitating the **interpretability** and communicability of the **intensional meaning** of the statement. Finally, the sender and the receiver must also have the same referential lexical competencies regarding the terms used in their communication. This is necessary to correctly designate and recognize their referents (i.e., extensional meaning). They must also share the same conventions for correctly placing statements in a **context** for their **actionability**.

#### Parallels between the structure of natural language statements and machine-readable data

The parallels between the structure underlying natural language statements about research findings (e.g., empirical observations, hypotheses, method descriptions) and corresponding (meta)data structures and their associated schemata become clear when considering that both are models representing a specific referent system.

A model can be understood as information on something (i.e., meaning) created by someone (i.e., sender) for somebody (i.e., receiver) for some purpose (i.e., usage context)^53^. The purpose of a model thereby is its use in place of the system it models—any answer that the model gives should be the same as what the referent system would provide, restricting the model to those properties of the system that are relevant for the purpose^54^. A model must possess the following three features^55^:

1. **mapping feature**: a model is based on a referent system, which it attempts to model;
2. **reduction feature**: a model only reflects a relevant selection of the properties of its referent system–no abstraction, no model; and
3. **pragmatic feature**: a model needs to be usable in place of its referent system with respect to a specific purpose.

By characterizing models in this manner, we can understand both **syntax trees with semantic roles** and **data structures** that are used in databases and data applications as **models**.

**Fig. 4:**
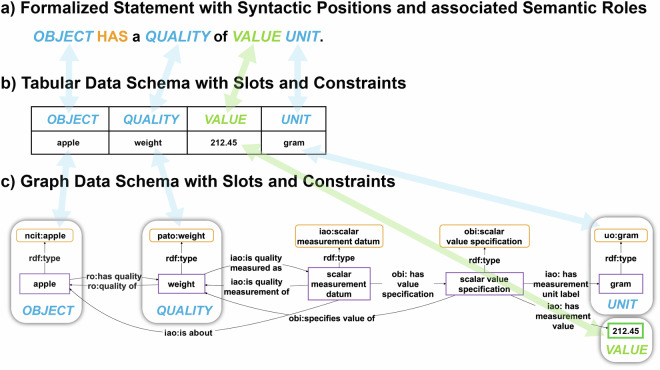
Parallels between natural language statements and data schemata. A data schema, both tabular as in (**b)** and graph-based as in (**c)**, must represent the same syntactic positions as its underlying formalized natural language statement in (**a)**. In a data schema, the syntactic positions take the form of slots, and each slot must specify its associated semantic role in the form of a constraint specification (constraint specifications not shown).

Token models can be distinguished from type models^54^. A **token model** (also called *snapshot model*, *representation model*, or *instance model*) models individual properties of elements of its referent system and is thus based on a one-to-one correspondence between the model and the system, representing the system’s individual attribute values such as the weight of a particular apple, with this apple being the referent system. **Token models** thus **model instances and their relations** and their referent systems are particular entities (or groups of particular entities). Therefore, the creation of a token model involves only **projection** (i.e., choice of properties to be modelled) and **translation** (i.e., translating the properties to model elements). The sentence in Fig. 2a, and the table and graph representation of the same information in Fig. 4b,c are examples for token models. Whereas the sentence is structured by English syntax and grammar, a table provides the structure for the table representation and a graph pattern the structure for the graph representation of this information. The elements in a token model designate the corresponding elements of the referent system—here, a particular apple and its particular weight. As a consequence, different token models of the same referent system that model the same system properties relate to each other through a transitive token-model-of relationship that can be ordered to chains of designators, each linearly designating its corresponding element across all token models, ultimately designating the corresponding element in the referent system^54^.

A **type model** (also called *schema model* or *universal model*), on the other hand, captures general aspects of a referent system through classification of its properties. Taking a look at the relation between a sentence (Fig. 2a), its corresponding syntax tree (Fig. 2b) and formalized statement (Fig. 2c), we see that the semantic role of a given syntactic position can be obtained by classifying the object or subject instance of the sentence to a specific type or class (*‘this apple’* instance → *‘apple’* class), which in turn can be generalized to a corresponding semantic role (*‘apple’* class → *‘OBJECT’* role). Creating a type model thus involves **classification** in addition to projection and translation. Formalized statements with syntactic positions and their associated semantic roles (Fig. 2c) are examples of type models and can be understood as **linguistic (data) schemata**. By classifying the allowed entities of a certain subject and object position, a sentence (i.e., token model) turns into a formalized statement type (i.e., type model) and thus a linguistic schema that specifies how a specific type of information should be communicated. Graph pattern specifications in the form of shapes provide the required structure for representing data in a knowledge graph and table specifications provide the corresponding structure for a relational database. Both specifications represent type models, where the constraint of a specific slot or column specifies the class of allowed instances.

Instantiating a type model produces a corresponding token model. This allows us, in turn, to validate a token model against its corresponding type model. We can say that the sentence *‘This apple has a weight of 212.45 grams’* is a token model (a datum) that instantiates the type model *‘OBJECT HAS a QUALITY of VALUE UNIT’* (a data schema) against which it can be validated. Applying different type models for modeling a given proposition, on the other hand, results in the creation of different token models of that same proposition (cf. Figure 3).

According to Kühne^54^, some type models are metamodels. A **metamodel** is a model of a model. In addition to projection, translation, and classification, metamodels involve **generalization**. As mentioned above, obtaining the semantic role of a syntactic position in a statement involves classification and generalization. Metamodels are more broadly applicable due to this generalization. Consequently, the formalized statement *‘OBJECT HAS a QUALITY of VALUE UNIT’* is a metamodel, as it is derived from the type model *‘APPLE HAS a WEIGHT of VALUE MASS-UNIT’* by generalizing the *‘APPLE’* slot into an *‘OBJECT’*, the *‘WEIGHT’* slot into a *‘QUALITY’*, and the *‘MASS-UNIT’* slot into a *‘UNIT’* slot. Formalized statements (cf. Fig. 2c) that model types of statements can thus be considered to be metamodels.

A metamodel *A* is defined by being a type model that relates to another model *C* via a type model *B*, where the relation-chain from *A* via *B* to *C* is through two type-model-of relations. Most (meta)data schemata are based on such metamodels, and when using language, we interpret sentences by using corresponding metamodels (see formalized statements in Fig. 2c and Fig. 3b). The structure used for documenting a datum either as a table in a relational database or a graph in a knowledge graph, is a metamodel and is defined through the specification of the table structure and the graph pattern, respectively (e.g., formal statement, CSV template, or a semantic schema specification using the Shapes Constraint Language SHACL^56^).

Analog to the characterization of sentences as token models and their corresponding formalized statements as type/meta models, we can understand a **proper name** to refer to an instance (i.e., individual) as its referent system, by designating a **token model** of that system that specifies an ontological/intensional definition and operational recognition criteria that refer to properties of that system. A **general term**, in contrast, refers to a class or concept by designating a **type/meta model** that specifies an ontological/intensional definition and operational recognition criteria that refer to properties of all instances of that class or concept.

Thus, if we understand the sentence *‘This apple has a weight of 212.45 grams’* to be a natural language token model of a corresponding real apple, modeling the properties of that apple via corresponding syntactic positions, then we can conclude that each slot of a (meta)data model of the same real apple that models the same set of properties is a token model that relates to the corresponding syntactic position of the language model through a transitive token-model-of relationship. Consequently, comparing natural language and (meta)data representations of the same referent system should be straightforward if they both model the same set of properties of the same referent system.

Therefore, we can think of each datum as a somewhat formalized representation of a natural language statement, structured in such a way that it can be easily compared with statements of the same type, and easily read and operationalized by machines (cf. Fig. 4a with Fig. 4b,c). A datum is a token model that results from the instantiation of a (meta)data schema that is its corresponding meta model. Both can be understood to be formalizations of a particular type of natural language token and meta model that serve the purpose to support both machine-actionability and human-interpretability. In other words, (meta)data schemata are to machines what syntax trees are to humans—both define meta models with positions and associated semantic roles for statements. They specify the structure of the corresponding data representation. When we compare (meta)data schemata with their corresponding natural language statements, we can thus see similarities between the structure of a sentence defined by the syntax and grammar of a natural language such as English and the structure of a corresponding schema (Figs. 2, 4). As discussed above, the syntactic positions of terms in a natural language sentence take on specific semantic roles and contribute significantly to the meaning of the statement.

For a (meta)datum to have the same meaning as its corresponding natural language statement, it is **necessary** that its underlying structure be functionally and semantically similar to the structure of the corresponding natural language statement. That is to say, the elements of the (meta)datum must comprise the same elements as the corresponding syntax tree: the schema must represent all relevant **syntactic positions**—in schemata often called **slots**—and their associated **semantic roles** in the form of **constraint specifications**, with terms and values populating the slots (see Fig. 4b,c). After all, humans need to be able to understand these (meta)data schemata and need to be able to translate a given datum represented in a given (meta)data schema back into a natural language statement. Following Guizzardi^32^, (meta)data are “*instruments used by humans to harmonize their conceptualizations and, hence, interoperability approaches succeed to the extent that they can safely connect these conceptualizations*” (p. 182). If the column headers in tabular (meta)data structures are not properly characterizing the semantic roles associated with their respective syntactic positions (e.g., *‘Location1’* and *‘Location2’* instead of *‘Departure Location’* and *‘Destination Location’* for passenger transport data), humans will have difficulties interpreting them correctly. (Meta)Data schemata should therefore be seen as attempts to translate the structure of natural language sentences into machine-actionable (meta)data structures.

### Towards a conceptual model of semantic interoperability

**Fig. 5:**
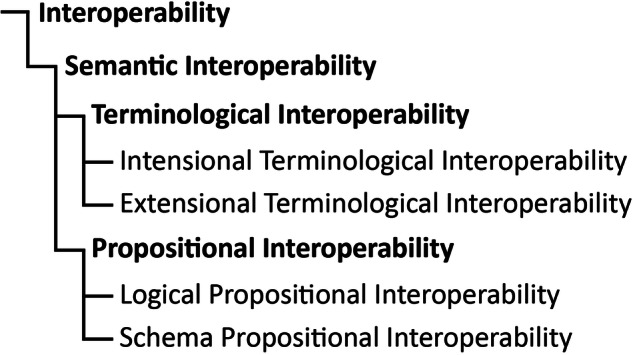
Hierarchy of different types of semantic interoperability.

Based on the findings from the **linguistic perspective on semantic interoperability** presented in the previous chapter, we can now contribute to a **conceptual model of semantic interoperability** by identifying and defining different **types of semantic interoperability** and their **hierarchical relationships** (see Fig. 5). In the introduction, we argued that the interoperability of a dataset is its disposition to take a specific role in a specific operation that can be executed by either a machine or a human being, requiring the dataset to be human-interpretable as a minimum requirement for its interoperability. Based on our findings from above, we now understand semantic interoperability of a dataset as a specific kind of interoperability that has ‘**communicating information**’ as its specific operation, with the dataset taking the role of the information carrying unit and either machines or humans taking the role of the information sender and receiver. The primary emphasis of general interoperability of a dataset is on its machine- and human-actionability, while the focus of semantic interoperability is on its machine- and human-interpretability. Since actionability depends on interpretability (see definitions above), general interoperability is dependent on semantic interoperability—if a dataset is not semantically interoperable ( = interpretable), it is not interoperable ( = actionable) at all. And the set of all semantically interoperable datasets is the combination of all machine-interpretable and all human-interpretable datasets.

In the previous chapter, we identified terms and statements as two categories of textual representational artifacts that take the role of foundational meaning-carrying units of information in human textual communication (see also Fig. 1). Consequently, we can distinguish **terminological interoperability** as the semantic interoperability of terms from **propositional interoperability** as the semantic interoperability of statements as two distinct types of semantic interoperability. In the following we differentiate subtypes of each of them based on the different types of possible semantic relationships between two terms and between two statements.

#### Terminological interoperability

Terminological interoperability refers to the semantic interoperability of terms. Terms as textual representational artifacts are used to identify or represent referent systems (e.g., real-world concepts or instances) and can be represented in (meta)data in the form of resources or literals that take the role of a value in a slot of a (meta)data structure. When comparing two given terms regarding their semantic content (cf. Fig. 1), we distinguish the following possible **semantic interoperability relationships** between them^24^ (Fig. 5):

1. **no terminological interoperability**: the two terms differ both in their intensional meaning and their referent/extension, as in ‘*apple*’ and ‘*car*’;
2. **extensional terminological interoperability**: they differ only in their intensional meaning but share the same referent, as in ‘*Morning Star*’ and ‘*Evening Star*’, which both refer to the planet Venus but differ in their intensional definitions, or in ‘*Pluto*_*dwarf planet*_’ and ‘*Pluto*_*planet*_’, which both refer to the same astronomical body from the solar system but differ in their ontological definition;
3. **intensional terminological interoperability**: they share the same intensional meaning and the same referent/extension; or
4. **other relations of semantic interoperability**: they differ in both their intensional meaning and their referent/extension, but some intensional and extensional overlap, closeness, or similarity relationship exists between them, as in ‘*apple*’ and ‘*fruit*’.

If two terms share their intensional meaning and their referent/extension, they are **extensionally and intensionally interoperable**, i.e., they are **strict synonyms** and can be used interchangeably. Since no two terms can share their intensional meaning without also sharing their referent/extension, intensional terminological interoperability always implies extensional terminological interoperability, but not vice versa. Thus, if two terms have the same referent/extension but not the same intensional meaning, because controlled vocabularies may differ in their ontological commitments, their intensional terminological interoperability is violated, but not necessarily their **extensional terminological interoperability**, since both terms may still be used to refer to the same referent system. Two terms are extensionally terminologically interoperable if they refer to the same set of (real world) referent systems, independent of whether they also share the same intensional meaning. Consequently, **the set of intensionally interoperable terms is a subset of the set of extensionally interoperable terms**.

For example, the COVID-19 Vocabulary Ontology (*COVoC*) defines ‘*viruses*’ (NCBITaxon:10239) as a subclass of ‘*organism*’ (OBI:0100026), while the Virus Infectious Diseases Ontology (*vido*) defines ‘*virus*’ (NCBITaxon:10239) as a subclass of ‘*acellular self-replicating organic structure*’ (IDO:0002000), and thus as an object that is not an organism (*vido* also reuses ‘*organism*’ (OBI:0100026), but does not classify ‘*virus*’ (NCBITaxon:10239) as one of its subclasses)–these two terms are therefore not intensionally interoperable as their ontological definitions differ, even though they have the same referent (i.e., the same extension) and even the same identifier, since both ontologies have imported the ‘*Viruses*’ class (NCBITaxon:10239) from NCBITaxon.

Researchers sometimes disagree on the classification of a given referent system, or, when modelling the same referent system, focus on different aspects of that system (cf. Fig. 1). As a consequence, they have different cognitive representations in the form of different concepts in mind, but still refer to the same thing as the referent system. In all these cases, they thus agree on the referent system but disagree on its ontological or intensional definition, i.e., the corresponding concept’s intensional meaning. Sometimes, researchers also change their mind due to new insights and change the classification of a given entity and thus change how they model it. Pluto is a good example of the latter, which has been recently re-classified from planet to dwarf planet. We can therefore distinguish Pluto_*dwarf planet*_ and Pluto_*planet*_, which are two different models that both refer to the same astronomical body from the solar system. Changes in conceptualization of a given (type of) referent system are common in empirical research, where some phenomenon needs an explanation, and in the course of research, new theories emerge that improve our understanding of the phenomenon. Each new theory provides a new ontological definition, i.e., an updated model, and the updated model and all precursor models share the same referent.

With respect to the matter of terminological interoperability, it can thus be conclude that, while intensional terminological interoperability is preferable, **extensional terminological interoperability constitutes the minimum requirement for the semantic interoperability of two terms**. This is due to the fact that, during the process of communication, it is essential to ensure that both the sender and the receiver know that they refer to the same (real) referent system.

In addition to these two clearly demarcated cases with actionable consequences for terminological interoperability, we can recognize several intermediate relations between terms that neither share their intensional meaning nor their referent/extension, reaching from no semantic relatedness to sharing some overlap, closeness, or similarity in their intensional meaning. While indicating the type of semantic relationship between them may represent useful information, it is not directly actionable in the context of terminological interoperability.

In the context of knowledge graphs and ontologies, if two terms share the same intensional meaning and the same referent/extension despite having different GUPRIs, we can express their terminological interoperability by specifying a corresponding **entity mapping** using the ‘*same as*’ (owl:sameAs; skos:exactMatch) property. This is straightforward for mappings across terms that refer to individuals, indicating that the mapped resources actually refer to the same individual entities. In OWL Full, however, where classes can be treated as instances of (meta)classes, it can also be used to specify that the two mapped classes not only have the same class extension and thus the same referent (which can be expressed using owl:equivalentClass) but also the same intensional meaning^57^. If two terms have only the same referent/extension but not the same intensional meaning, we can express their extensional terminological interoperability by specifying a corresponding entity mapping using the ‘*equivalent class*’ (owl:equivalentClass) property. **We can thus distinguish between intensional (i.e., same-as) and extensional (i.e., equivalent-class) entity mappings**. Both types of mappings are homogeneous definition mappings^58^, where there is only one vocabulary element to be mapped on the left side, and several others on the right side of the definition that do not need to be mapped. Additionally, entity mappings can document further relationships, which can be helpful in solving interoperability issues (see Table 1). With the Simple Standard for Sharing Ontological Mappings (**SSSOM**)^59–61^, an established standard exists for documenting these entity mappings.

**Table 1:** A table of properties that can be used to specify terminological interoperability relations between two terms in an entity mapping and their applications. The property indicated by * is a property we suggest for specifying extensional entity mappings in case owl:equivalentClass should not be used.

entity mapping relation	application
owl:sameAs; skos:exactMatch	A transitive and symmetrical relation between two resources (i.e., terms) that are either concepts referring to an individual or instance (i.e., proper name) or to a class (i.e., general term or kind term), where both terms share the same intensional meaning and the same referent/extension. We suggest using them for indicating intensional terminological interoperability between concepts. *Example: uberon:multicellularOrganism (UBERON:0000468) and ocimido:multicellularOrganism (OCIMIDO:00467)*.
owl:equivalentClass; *new:extensionalMatch*	A transitive and symmetrical relation between two resources (i.e., terms) that are either concepts referring to an individual or instance (i.e., proper name) or to a class (i.e., general term or kind term), where both terms share the same referent/extension but not necessarily also the same intensional meaning. We suggest using them for indicating extensional terminological interoperability between concepts. *Example: uberon:multicellularOrganism (UBERON:0000468) and caro:multicellularOrganism (CARO:0000012)*.
owl:equivalentProperty	A transitive and symmetrical relation between two properties, where both properties share the same extension but not necessarily also the same intensional meaning. We suggest using them for indicating extensional terminological interoperability between properties. *Example: dcat:hasVersion and pav:hasVersion*.
rdfs:subClassOf	A transitive relation between two classes, where the domain (i.e., subject) specifies the parent class and the range (i.e., object) the subclass. *Example:* foodon:animal (FOODON:00003004) and *uberon:multicellularOrganism (UBERON:0000468)*.
rdfs:subPropertyOf	A transitive relation between two properties, where the domain (i.e., subject) specifies the parent property and the range (i.e., object) the subproperty. *Example: ro:hasComponent (RO:0002180) and bfo:hasPart (BFO:0000051)*.
skos:closeMatch	A non-transitive, symmetrical relation between two concepts (either instances or classes), indicating that they are sufficiently similar to be used interchangeably in some information retrieval applications. This relation may be of value to human readers, but it is not defined by formal semantics and thus cannot be used by machines in any meaningful sense.
skos:relatedMatch	A non-transitive, symmetrical relation between two concepts (either instances or classes), indicating an associative relation between them. This relation may be of value to human readers, but it is not defined by formal semantics and thus cannot be used by machines in any meaningful sense.
skos:broadMatch	A non-transitive, hierarchical relation between two concepts (either instances or classes), indicating that the object is broader than the subject. This relation may be of value to human readers, but it is not defined by formal semantics and thus cannot be used by machines in any meaningful sense.
skos:narrowMatch	The inverse relation of skos:broadMatch.

In this context, we want to note that practically achieving intensional terminological interoperability between class terms across different ontologies often requires evaluating the terminological interoperability between all terms referenced in the respective class axioms (i.e. the formal semantic definitions of the class terms). If the schemata underlying the axioms differ, schema crosswalks must be specified (see propositional interoperability below). If the class terms referenced in class axioms differ across class axioms, entity mappings must be specified for them as well, provided they have the same intensional meaning and the same referent/extension, before the semantic interoperability relation between the class terms can be identified and respective entity mappings be specified. This is necessary because the intensional meaning of a term is conveyed by its intensional and/or ontological definitions, which are statements in their own right, with all the resulting consequences for propositional interoperability. Unfortunately, however, this propositional aspect of terminological interoperability is often overlooked.

### Propositional interoperability

Propositional interoperability refers to the semantic interoperability of statements. Statements as textual representational artifacts are used to identify or represent relationships between referent systems and can be represented in a (meta)data structure in the form of several resources or literals taking the roles of values in different slots within that structure. When comparing two given (meta)data statements regarding their semantic content, we distinguish the following possible **semantic interoperability relationships** between them^24^ (Fig. 5):

1. **no propositional interoperability**: the two statements model different types of statements and thus different types of information, as in a volume measurement statement and a universal causal relationship statement, and the corresponding (meta)data statements are modelled based on different logical frameworks;
2. **logical propositional interoperability**: they may or may not model different types of statements and thus different types of information, but they are both modelled based on the same logical framework so that reasoning operations can be applied on them when combined;
3. **schema propositional interoperability**: they model the same type of information and use the same (meta)data schema so that machines can automatically align slots with the same associated semantic roles; or
4. **other relations of semantic interoperability**: they model different types of statements and thus different types of information, but some intensional and extensional overlap, closeness, or similarity relationship exists between them.

**Logical propositional interoperability** between two (meta)data statements is achieved if both statements are modeled on the basis of the same logical framework (e.g., OWL2-DL) and this information has been made explicit in the statement’s metadata, so that one can reason over the combination of both statements using appropriate reasoners (e.g., Pellet^62^). It is important to realize that logical propositional interoperability and logical consistency are two different data characteristics: logical propositional interoperability only depends on whether **reasoning operations** can be applied to the data, and does not depend on their logical consistency.

In the context of ontologies and FAIR knowledge graphs, we can also talk about the logical propositional interoperability between class terms in reference to their ontological/intensional definitions in the form of class axioms, which are universal statements that are logically propositionally interoperable if they are represented using the same logical framework.

**Fig. 6:**
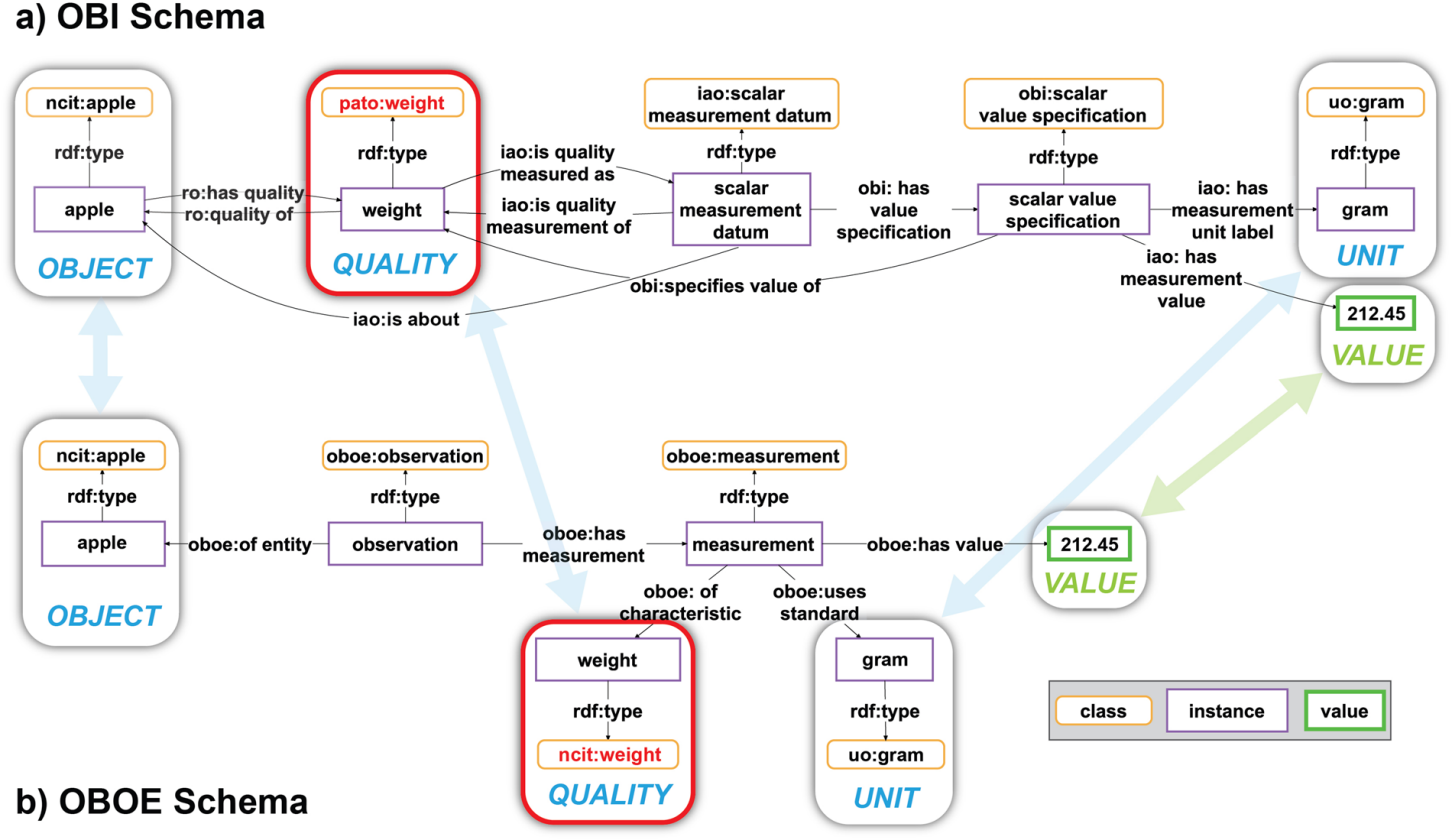
Crosswalk from one schema to another for a weight measurement statement. The same weight measurement statement is modeled using two different schemata. (**a)** The weight measurement according to the schema of the Ontology for Biomedical Investigations (OBI)^76^ of the Open Biological and Biomedical Ontology (OBO) Foundry, which is often used in the biomedical domain. (**b)** The same weight measurement statement according to the schema of the Extensible Observation Ontology (OBOE), which is often used in the ecology community. The arrows indicate the alignment of slots that share the same constraint specification, i.e., the same semantic role. The corresponding semantic roles include the *OBJECT*, the *QUALITY*, and the *VALUE* that has been measured together with its *UNIT*. The slots carry the information that conveys the intensional meaning of the weight measurement statement to a human reader. Blue arrows indicate slots with resources as values, and green arrows those with literals. Slots with red borders indicate problems with terminological interoperability: OBO uses an instance of the class ‘pato:weight’, while OBOE, in this example, uses an instance of the class ‘ncit:weight’. However, since ‘pato:weight’ and ‘ncit:weight’ are synonymous terms and can therefore be mapped as ‘skos:exactMatch’, the problem can be resolved with the corresponding intensional entity mapping. While this figure illustrates a schema crosswalk between two semantic data schemata, schema crosswalks can also be used to establish schema propositional interoperability between two relational (meta)data schemata or between a relational and a semantic (meta)data schema (e.g., see arrows in Fig. 4).

**Schema propositional interoperability** between two (meta)data statements is achieved when they represent statements of the same type and are documented using the same (meta)data schema. If statements of the same type were represented using different schemata, corresponding semantic contents would no longer be semantically interoperable. In such cases, one would have to specify schema crosswalks (i.e., schema mappings) to regain schema propositional interoperability (see Fig. 6).

A **schema crosswalk** constitutes a set of rules that specify the alignment of (meta)data elements or attributes and thus slots in one schema and format (e.g., syntactic positions and tables or columns in tabular data formats) with the equivalent slots in another schema and format. Equivalent slots share the same constraint specifications (i.e., the same semantic role). The constraint specifications of aligned slots are mapped to each other using entity mappings. The objective of a schema crosswalk is to enable the transfer of a (meta)data statement between two given schemata. In other words, to achieve schema propositional interoperability between two given statements of the same statement type, the subject, predicate, and object slots of their corresponding (meta)data schemata need to be aligned, and their terms need to be mapped across the controlled vocabularies used in each aligned slot. Schema crosswalks thus go beyond entity mappings, as they do not merely map one individual term to another but align slots for terms based on their semantic roles. Consequently, we need specific minimum metadata standards for schema crosswalks in addition to standards such as the SSSOM for entity mappings.

The use of schema crosswalks can be compared to how we recognize different natural language sentences as different expressions of the same underlying proposition (cf. Fig. 3). If the schemata used by two (meta)data statements use different vocabularies to populate their slots (i.e., the constraint specifications of their slots refer to different vocabularies, the values in the cells of tabular (meta)data formats use different vocabularies), then corresponding entity mappings must be included in the crosswalk to ensure terminological interoperability (see red bordered slots in Fig. 6). Consequently, schema propositional interoperability is dependent on the terminological interoperability of the terms used in the specifications of their slot constraints. Since schemata typically comprise multiple slots, we can distinguish **intensional and extensional schema crosswalks** as two poles defining the continuum of possible schema crosswalks, with the former requiring no or only intensional entity mappings, and the latter requiring for each slot an extensional entity mapping.

In the context of ontologies and FAIR knowledge graphs, statements as machine-actionable information take the form of either ABox semantic instance-graphs and thus assertional statements or TBox semantic graphs in the form of class axioms and thus universal statements, and they must be modelled based on a formally specified semantic (meta)data schema, e.g., in the form of graph patterns specified as SHACL shapes. Shapes that share the same statement-type as their referent then need to be aligned and mapped. These are **type/meta model alignments** (i.e., ontology pattern alignments; TBox)^58^ or **token model alignments** (i.e., ABox), where several vocabulary elements must first be aligned and then mapped in schema crosswalks. If two schemata share the same constraints across all of their slots (i.e., any statement expressed in schema *A* can be expressed in schema *B* using the same set of terms without violating their slot-constraints), the corresponding crosswalk can specify them as identical regarding their intensional meaning (analogue to entity mappings, via owl:sameAs or skos:exactMatch). All other crosswalks can be specified as extensionally identical (via owl:equivalentClass) or as semantically overlapping or semantically similar using appropriate properties.

As far as propositional interoperability is concerned, we can therefore conclude that although a combination of logical propositional interoperability and intensional schema propositional interoperability is preferred, for the same reasons as for terms, **schema propositional interoperability using extensional schema crosswalks constitutes the minimum requirement for the semantic interoperability of two statements**.

Regarding the concept of *model* as discussed further above, entity mappings and schema crosswalks both represent information on the transformation of a source to a target model and are thus models of model-to-model transformations with the purpose to automate the translation process (on transformations as model see^54^). Such a transformation only translates those properties (i.e., syntax positions with associated semantic roles or slots with constraints) of the source to the target model that are relevant for the target model’s purpose. Entity mappings are modelling the transformation between token models (i.e., between two proper names) or between type models (i.e., between two kind or general terms) of specific referent systems, whereas schema crosswalks are modelling the transformation between two type models of the same referent statement type.

In summary, the semantic interoperability of two given datasets is directly dependent on the completeness of readily available intensional and extensional entity mappings and schema crosswalks for all the terms and statements they comprise.

### How to achieve semantic interoperability: What makes a term a good term and a schema a good schema?

One might posit that the achievement of general semantic interoperability of (meta)data across the Web is contingent upon the development of a common standard, format, and schema for each type of referent entity and each type of statement to which every (meta)data provider and consumer must agree on. This standard and format must be as rich as the constituent system models. In other words, the goal would be to achieve full semantic harmonization across all (meta)data sources, requiring convincing all stakeholders to agree on the common standard. This **integrated interoperability**^63^ approach to achieving interoperability is presumably one of the reasons for the proliferation of standards that we see in so many areas.

For terms, this approach would require agreement on a universal terminology. This, however, is not feasible. Even when ignoring societal, psychological, and historical factors, different research communities often apply different **frames of reference** and thus emphasize different aspects of a given referent system they want to model, resulting in the need for different terms for the same type of system, inevitably resulting in issues with semantic interoperability. For instance, for some studies and experiments, an ontology that is based on quantum mechanics, in which an electron is both a particle and a wave, would not be adequate for modelling the aspects of reality relevant to the experiment and, instead, an ontology that is based on Newtonian physics, in which an electron is only a particle, would be preferable. Consequently, there is sometimes a **legitimate demand for more than one term for a particular referent system**—a fact that is in direct opposition to the goal of a universal terminology.

The situation for schemata is similar. A good schema for a (meta)data statement must cover all the information that needs to be documented, stored, and represented for that type of statement. However, there are many other criteria for evaluating schemata. Most of these are related to the **different operations** one wants to perform on the (meta)data. The choice of schema depends on the underlying purpose of a study, which in turn determines which operations will be performed on the (meta)data. Each operation likely has different requirements on the schema in terms of performance optimization. Moreover, format requirements from corresponding tools and thus demands of **fitness-for-use**, allowing direct use of (meta)data, also influence the overall degree of machine-actionability of the (meta)data, and thus the choice of a (meta)data schema for a given study. Optimizing the findability of measurement data, for instance, likely requires a different data schema than optimizing reasoning over them. A given schema must therefore be evaluated in terms of the operations to be performed and the tools to be used on the (meta)data, often involving trade-offs between different operations with different priorities to achieve an overall optimum.

Therefore, although agreement on a universal terminology and a universal set of schemata would be a solution for achieving semantic interoperability and machine-actionability of (meta)data across different research domains, this is unlikely to happen for the reasons discussed above, and **we have to think pragmatically** and emphasize the need for intensional and extensional entity mappings for terminological interoperability and schema crosswalks for schema propositional interoperability. As a result, integrated interoperability is not a viable approach to achieving interoperability in the sense of FAIR.

**Fig. 7:**
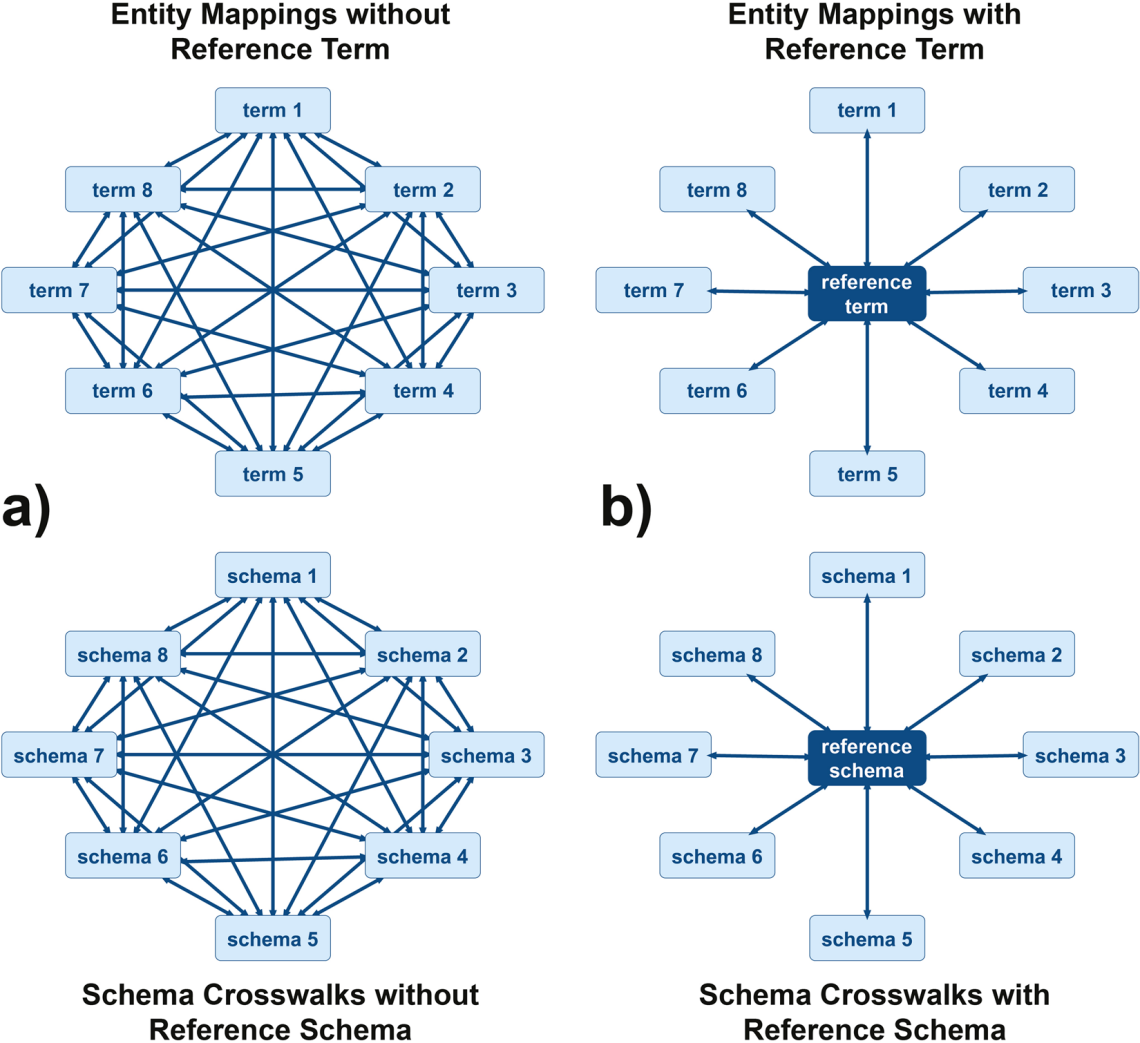
Comparison of number of required entity mappings and schema crosswalks with and without a reference term or reference schema. (**a)** 28 entity mappings and schema crosswalks respectively are required to achieve terminological and schema propositional interoperability between 8 different terms and schemata, because each possible pair of terms and schemata requires its own mapping and crosswalk. (**b)** With a reference term and a reference schema playing the role of an intermediary, the number of required mappings and schema crosswalks can be reduced to a minimum of only 8, which significantly reduces the effort required to establish terminological and schema propositional interoperability for the corresponding type of term and type of (meta)data statement.

Alternatively, the **unified interoperability** approach has been suggested^63^, according to which semantic interoperability is achieved by agreeing on a common meta-level structure for establishing comparable representations of diverse formats and schemata via super metamodels. If a **super metamodel** attained widespread adoption as a reference, various formats and schemata could be mapped to it, allowing (meta)data conversion across these formats and schemata using the super metamodel as mediating structure, achieving semantic interoperability across all formats and schemata (see, e.g., *reference term* and *reference schema* in Fig. 7). Examples of this approach are the Cross Domain Interoperability Framework that is intended to be used as a *lingua franca* for (meta)data sharing across domain boundaries, LinkML as a *lingua franca* for different schema modeling languages, allowing the export of a LinkML schema to other representations, and I-ADOPT that is aimed at unifying the semantic description of research variables.

The **federated interoperability** approach^63^, on the other hand, assumes that (meta)data formats and schemata have to be adapted dynamically instead of having a predefined super metamodel. A **syntactic variant** of federated interoperability has been suggested, in which multiple point-to-point conversions are required that are achieved by specifying *n*^2^ entity mappings and schema crosswalks for federating *n* alternative terms and schemata. Ontology mappings typically take the form of such point-to-point entity mappings.

Sadeghi *et al*.^36^ suggest that the syntactic variant could be replaced by a **semantic variant** of federated interoperability that would provide meaning of (meta)data structures in a machine-interpretable way through the use of one or more (foundational) ontologies. They argue that ontologies would provide agreed meaning of the concepts used in the (meta)data structures, resulting in a scalable approach, requiring only mappings to ontology resources, reducing the number of required mappings to *n* for federating *n* alternative terms, instead of *n*^*2*^ as in the syntactic variant. However, if the semantic variant presupposes a semantic abstraction layer to which each system can map and crosswalk their (meta)data and artefacts, then this approach becomes equivalent with the unified interoperability approach, with the semantic abstraction layer taking the role of the super metamodel. Above, we already discussed why ontologies and semantic (meta)data schemata cannot serve this purpose. Moreover, the authors mention only entity mappings and do not explain how this approach achieves propositional interoperability.

In any case, one can understand a term or a statement as a model of a referent system that has been developed with a particular purpose in mind. If we consider that this always involves a certain degree of abstraction and reduction (see *reduction feature* of a model), it becomes clear that for some referent systems we need more than one model. This is especially the case for all real-world referent systems, which are typically rich, comprising a potentially infinite number of properties, each of which may be relevant in a particular context. The reduction feature of a model, and thus the choice of which properties one considers to be relevant for a specific purpose, specifies the model’s **ontological commitment**. With respect to semantic interoperability, it is therefore important that a model’s ontological commitment is clearly documented.

We concur with Guizzardi^32^ that the existence of good ontologies in general, and foundational ontologies in particular, can provide formal, shared, and explicit representations of fundamental conceptualizations. These conceptualizations can then be used for the documentation of (meta)data. Guizzardi asserts that achieving semantic interoperability for machines is contingent upon ontologically consistent conceptualizations (i.e., models), which are only provided by foundational ontologies. We agree with Guizzardi, but would like to add that (foundational) semantic (meta)data schemata must also be provided, since ontologies only model what is necessarily the case for each instance of a particular type of entity, expressed as universal statements, but not what is actually the case for each individual entity, expressed as assertional statements, or what can be or is typically the case, expressed as contingent or prototypical statements.

It is noteworthy that in domains pertaining to machine-actionability and dealing with highly heterogeneous (meta)data, such as Industry 4.0 and health with their electronic-health-record-based systems, ontologies and conceptual models based on OWL are used as the most recurrent solutions^21^. We are not suggesting that (foundational) ontologies, OWL, and semantic (meta)data schemata are the sole solution for achieving machine-actionable semantic interoperability; however, they appear to be the most promising solution at this time.

### FAIR 2.0: Extending the FAIR Guiding Principles

As demonstrated above, achieving semantic interoperability is a complex task, and establishing the IFDS is therefore a significant challenge that requires more than merely organizing (meta)data into FAIR Digital Objects and specifying mappings across terms for achieving terminological interoperability. To date, discourse on semantic interoperability and the associated tools and services has predominantly centered on terminological interoperability, focusing mainly on entity mappings across various controlled vocabularies and ontologies, and a strong emphasis on basic metadata. This is not surprising, considering that solutions for propositional interoperability must build on solutions for terminological interoperability, and basic metadata can be modelled domain-independently. Moreover, whereas a term (in the broad sense, including literals) can be formally conceptualized as a resource represented by a GUPRI or a literal with associated datatype specification and thus as a clearly specified and demarcated entity, specifying and demarcating a (meta)data statement is not as straightforward. When modelled in a table, a (meta)data statement can consist of more than two columns and when modelled as a graph, of one or more triples (e.g., Fig. 4b,c). Since we currently lack agreed upon semantic categories at granularity levels coarser than terms or single triples, it is not obvious how to conceptualize and clearly demarcate (meta)data statements. However, because information and meaning are communicated through such statements and not through individual terms, we need to understand statements (i.e., propositions) as a basic unit of information, and we need to find a solution for achieving propositional interoperability for them to obtain FAIR (meta)data. And in a next step, we must think about how to make collections of statements interoperable. We thus need, in addition to controlled vocabularies, ontologies, and entity mappings for terminological interoperability also something analogous for statement types and collections of statements.

Statement types are characterized by their underlying main verb or predicate. All statements about the weight of a particular object belong to the weight measurement statement type, and all statements about parthood relations between objects to the parthood statement type. It must be clarified what is required for statements and collections of statements to be FAIR.

We believe that the FAIR Guiding Principles need to be extended to cover the FAIRness of statements and collections of statements (see also Table 2). Above, we argue that the interoperability of a given dataset in terms of its machine- and human-actionability directly depends on its semantic interoperability and thus the machine- and human-interpretability of all terms and statements it contains. The initial step in assessing the overall interoperability of a dataset—and with it also its degree of FAIRness—is therefore to evaluate its semantic interoperability, which, however, requires specifying the semantic relationships between all terms and statement types the dataset covers.

**Table 2:** The updated FAIR Guiding Principles and their association with layers of the EOSC Interoperability Framework (EOSC IF) and the different aspects of semantic interoperability discussed above, including the original FAIR Guiding Principles^5^ (in regular font) and proposed additions (in bold font) towards a FAIR 2.0 Guiding Principles.

The Updated FAIR Guiding Principles	EOSC IF
**To be Findable:**	
F1. (meta)data are assigned a globally unique and persistent identifier	*technical*
F2. data are described with rich metadata (defined by R1 below)	*semantic*
F3. metadata clearly and explicitly include the identifier of the data it describes	*semantic*
F4. (meta)data are registered or indexed in a searchable resource	*technical*
**To be Accessible:**	
A1. (meta)data are retrievable by their identifier using a standardized communications protocol	*technical*
A1.1 the protocol is open, free, and universally implementable	*technical & legal*
A1.2 the protocol allows for an authentication and authorization procedure, where necessary	*technical*
**A1.3 the protocol is compliant with existing data protection regulations (e.g**., General Data Protection Regulation**, GDPR)**	***organisational & legal***
A2. metadata are accessible, even when the data are no longer available	*technical & organisational*
**To be Interoperable:**	
I1. (meta)data use a formal, accessible, shared, and broadly applicable language for knowledge representation	*semantic & technical*
I2. (meta)data use vocabularies that follow FAIR principles **to support terminological interoperability**	***semantic-terminological*** *& technical*
**I2.1 vocabularies used in (meta)data ideally include multilingual labels and specify all relevant synonyms for their terms**	***semantic-terminological***
**I2.2 terms with the same intensional meaning and/or the same extension are ideally mapped across all relevant vocabularies through intensional or extensional entity mappings**	***semantic-terminological***
**I2.3 terms provide human-readable specifications of their intensional meaning and, where applicable, human-readable recognition criteria for correctly applying them**	***semantic-terminological***
I3. (meta)data include qualified references to other (meta)data	*semantic*
**I4. (meta)data schemata are used that support propositional interoperability**	***semantic-propositional***
**I4.1 ideally, the same (meta)data schema is used for the same type of statement or collection of statement types, and the schema is referenced with its identifier in the statement’s metadata**	***semantic-propositional***
**I4.2 ideally, (meta)data schemata for the same type of statement are aligned and mapped across all relevant schemata (i.e., schema crosswalks)**	***semantic-propositional***
**I4.3 (meta)data use a formalism to clearly distinguish between lexical, assertional, contingent, prototypical, and universal statements**	***semantic-propositional***
**I4.4 (meta)data specify the logical framework that has been used for their modeling (e.g., Description Logics using OWL or First Order Logic using Common Logic Interchange Framework)**	***semantic-propositional***
**To be Reusable:**	
R1. (meta)data are richly described with a plurality of accurate and relevant attributes	*semantic*
R1.1 (meta)data are released with a clear and accessible data usage license	*legal*
R1.2 (meta)data are associated with detailed provenance	*semantic*
R1.3 (meta)data meet domain-relevant community standards	*semantic & organisational*
**R1.4 metadata indicate the certainty level of the truthfulness of the semantic content of their corresponding data**	***semantic***

FAIRness is not a Boolean property; rather, it is a disposition to take specific roles in various types of operations executed by both machines and humans. This disposition is what enables interoperability. **The FAIRness of a dataset does not describe an intrinsic property of that dataset. Rather, it describes a particular relationship between the dataset and a data ecosystem, as well as between the dataset and its potential user communities**. Therefore, assessing the FAIRness of a particular dataset must also involve the assessment of the vocabularies and schemata used by the dataset. The FAIR Guiding Principles must reflect this relationship. We therefore propose the following additional criteria to be incorporated into the FAIR framework to achieve comprehensive semantic interoperability. We have also added one criterion to the accessibility principle and one to the reusability principle, which we believe would contribute to an updated FAIR 2.0. While the remaining additions are to the interoperability principle, they also indirectly affect the findability and reusability, both of which are highly dependent on interoperability:

**A1. (Meta)Data retrievability**

- **A1.3**: To encompass organizational and legal interoperability, (meta)data must comply with existing data protection regulations, such as the General Data Protection Regulation (GDPR). By adhering to relevant data protection regulations, organizations can facilitate secure and compliant (meta)data sharing, promoting seamless collaboration and (meta)data utilization.
**I2. Terminological interoperability**
- **I2.1**: The need to use controlled vocabularies for the terms used in (meta)data that provide synonyms and language-specific labels for multiple languages.
- **I2.2**: The need to map terms with the same intensional meaning and/or the same extension to each other, effectively establishing entity mappings for terminological interoperability. These mappings must follow an established standard (e.g., SSSOM) and should differentiate intensional from extensional and other types of entity mappings. By ensuring entity mappings across relevant vocabularies, datasets can be seamlessly communicated and their information exchanged, fostering efficient (meta)data integration and analysis.
- **I2.3**: Vocabularies used for the documentation of (meta)data must specify an ontological/intensional definition that is human-readable. This definition characterizes the intensional meaning of a given concept as a textual representational artifact. To serve as FAIR vocabularies, method-dependent recognition criteria should be provided when appropriate, specifying how to identify the referent entity. The explicit specification of ontological/intensional definitions and recognition criteria is paramount in ensuring interpretability and actionability for human users. This facilitates not only the comprehension of the intensional meaning but also the successful application of terms in designation (i.e., object given, matching term sought) and recognition (i.e., term given, matching object sought) tasks.

**I4. Propositional interoperability**

- **I4.1**: Ideally, uniform (meta)data schemata are maintained for statements of the same type, while referencing the schema’s identifier in the statement’s metadata. In some cases, (meta)data comprise specific collections of statements (e.g., material data sheets that characterize a specific type of material following an established standard). In such cases, the identifier must reference a schema that is the collection of the corresponding individual statement schemata. By adhering to this principle, propositional interoperability is improved, as statements with consistent schemata can be efficiently queried and processed.
- **I4.2**: All (meta)data schemata relevant for statements of the same type are ideally aligned and mapped to each other in the form of schema crosswalks. By achieving propositional interoperability through schema crosswalks, datasets can effectively exchange information and ensure compatibility across various (meta)data representations.
- **I4.3**: (Meta)Data must use a formalism that clearly distinguishes between lexical, assertional, contingent, prototypical, and universal statements^26,64^. The two statements ‘*This swan is white*’ and ‘*All swans are white*’, despite using the same set of terms (e.g., *swan* = ’Cygnus’ (NCBITaxon:8867); *white* = ’white’ (PATO:0000323)), differ substantially in their semantic content. Distinguishing these different categories of statements is thus not only important for human readers for correctly interpreting the statements’ intensional and extensional meaning and thus contribute to their propositional interoperability, but it is essential for their machine-interpretability. Moreover, classifying statements along these five categories also contributes to their findability.
- **I4.4**: The logical framework used for modeling (meta)data must be specified. By explicitly stating the logical framework, such as Description Logics using OWL or First Order Logic using the Common Logic Interchange Framework, propositional interoperability is further promoted, enabling standardized (meta)data representations and query mechanisms.

**R1. Richly described (meta)data**

- **R1.4**: Ideally, metadata specify the certainty level (i.e., level of confidence) of their semantic content and thus the information that their data contains, which is essential for proper reuse of data and for preventing phenomena such as citation distortion^65,66^.

## Discussion

### FAIR services

At their heart, the FAIR Guiding Principles offer recommendations for achieving rich machine-actionable (meta)data. In the light of the aforementioned challenges concerning FAIRness and semantic interoperability, it is evident that establishing a standard for FDOs that is solely predicated on a set of minimum required metadata and a particular format will prove inadequate for achieving this objective and establishing the IFDS. As previously mentioned, interoperability and findability of a dataset are not Boolean properties. Therefore, the FAIRness of a dataset is not a Boolean property; rather, it is situated in a multidimensional FAIRness space. Consequently, characterizing a dataset as FAIR offers limited insights. Regarding the dataset’s usability, it would be more informative to ascertain its degree of overall semantic interoperability and machine-actionability. To assess the semantic interoperability of a dataset, it is necessary to specify the vocabularies and (meta)data schemata it employs. Based on this information, the findability of all (meta)data contained within the dataset can also be evaluated. To evaluate the machine-actionability of a dataset, the operations and tools that can readily be applied to it must be specified.

However, this information is not readily available and should not be directly linked to a dataset. Instead, the development of **FAIR Services** is necessary to provide this information and support the FAIRness of any given type of FDO by indicating which operations can be conducted on the (meta)data schemata and the vocabularies that it uses, and which functions must be added to the Services for operations that are not yet supported for this type of FDO. In accordance with the conceptual model of semantic interoperability outlined above, FAIR Services are expected to facilitate the establishment of an **interoperability assessment model**^21^. This model is designated to identify barriers and assess the achievement levels concerning various interoperability types, thereby providing a framework for enhancing the interoperability of (meta)data. By providing essential and readily usable interoperability tools, FAIR Services would substantially contribute to an **interoperability framework**^21^ that aims at supporting human users of (meta)data and developers of information management systems. It is crucial to understand that cognitive interoperability^24^ is central to the (re)usability of all (meta)data. We believe that such FAIR Services must comprise the following components:

1. A **terminology service** for supporting terminological interoperability. The service must comprise a repository and registry not only for controlled vocabularies, thesauri, taxonomies, and ontologies but also for entity mappings, with each mapping being documented as a FDO that can be referenced via its GUPRI and curated independent of any terminology. The entity mappings must distinguish intensional from extensional mappings and other types of mapping relations (see Table 1) and should follow standards with detailed metadata (e.g., SSSOM). The service must also include a look-up service for terms and for entity mappings.
2. A **schema service** for supporting propositional interoperability. It must comprise a repository and registry not only for all kinds of (meta)data schemata (covering graph-based, tabular, and other kinds of (meta)data structures), organized based on the statement type(s) that the schema models, but also for schema crosswalks. Each schema and each crosswalk must be documented as a FDO that can be referenced via its GUPRI and curated independent of any other schema or crosswalk. Both schemata and crosswalks should adhere to standards for their documentation that are comparable to the SSSOM standard. In a manner analogous to the terminology service, the schema service must additionally include a look-up service for schemata and schema crosswalks. Since schemata for the same type of statement frequently not only differ in the way they relate the different slots (i.e., syntactic positions) but also in the choice of vocabularies/ontologies for specifying constraints on slots (see Fig. 6), the schema service must utilize entity mappings and thus interact with the terminology service. The look-up service of the terminology service, in turn, can gather information from the schema service regarding which schemata can be used for a given term. Moreover, in order to operationally use schema crosswalks, the schema service must also interact with the operations service.
3. An **operations service** for providing operations on FDOs. The service must comprise a repository and registry for all kinds of functions (i.e., executable code) for operations that can be conducted on (meta)data, such as unit conversion and data analysis, but also converting (meta)data across different schemata using schema crosswalks and entity mappings. Each function must be documented as a FDO that can be referenced via its GUPRI and that can be associated with those schemata and vocabularies that it can be applied to by referencing their corresponding FDOs.

If, in addition to such FAIR Services, the **FDOs of (meta)data statements** or **of semantically meaningful collections of statements** covered the following metadata, we would have a FAIR ecosystem at our disposal that could achieve a high degree of FAIRness for all kinds of (meta)data, and could additionally be used for evaluating and specifying the FAIRness of a given dataset:

1. the metadata of the FDO references the GUPRI of the schema that has been applied to structure its content, with the schema itself being described, registered, and made available as a schema FDO in a schema repository;
2. the metadata differentiates between the creator of the FDO and the author(s) of its contents;
3. the metadata specifies the type of statement(s) the FDO contains (i.e., lexical, assertional, contingent, prototypical, universal);
4. the metadata specifies the formal logical framework that has been applied in the FDO, if any, to inform whether one can reason over its contents and which logical framework must be used for it;
5. the metadata specifies the degree of certainty of the contents of the FDO; and
6. the FDO provides a human-readable representation of its content to meet requirements of cognitive interoperability^24^.

Agreeing on a minimum metadata standard for FDOs would increase the **accessibility** of data in the IFDS. However, for the IFDS to be truly FAIR, FAIR Services would also have to provide the entity mappings and schema crosswalks operationally, supporting not only the applicability of operations across different schemata and terminologies (**interoperability**) and facilitating (meta)data integration and thus (meta)data **reuse** in the IFDS, but also increasing the general **findability** of (meta)data across different platforms and repositories in the IFDS, independent of the particular terminologies and (meta)data schemata used. As a result, **FDOs that are supported by FAIR Services as described above would represent units of increased interoperability within the IFDS**.

In order to implement FAIR Services in an effective manner, it is essential to take into consideration the existing and ongoing work that has been carried out in this field. This work can be reused and built upon whenever possible. For instance, a number of terminology services exist, some of which are registries, such as Linked Open Vocabularies (LOV), the BioRegistry or Archivo, that mostly provide metadata on the terminology level, while others are look-up services, such as EMBL-EBI Ontology Look-Up Servcie (OLS) and the TIB Terminology Service that is based on it, or NCBO BioPortal and related OntoPortal based domain repositories, that also provide metadata on the term level and additional features for browsing, displaying term mappings (e.g., generated by the LOOM algorithm), or managing the indexed terminologies. There is also Skosmos^67^, an open source web-based browser and publishing tool specialized for Simple Knowledge Organisation System (SKOS) vocabularies. The Basic Register of Thesauri, Ontologies & Classifications (BARTOC) is a database of knowledge organization systems and related registries which collected and lists over 100 terminology registries. Many of the latter are often focused on indexing terminologies of specific research areas only, such as Biology, Chemistry, or Medicine. Consequently, there is the need to use terminology metadata schema standards, such as the Metadata for Ontology Description and Publication Ontology (MOD), for the interoperability between all of these terminology services with regard to synchronizing terminology and mapping metadata for interdisciplinary contexts and use cases.

EMBL-EBI Ontology Xref Service (OxO)^68^ is a service designed to find mappings or cross-references between terms from various ontologies, vocabularies, and coding standards. OxO imports these mappings from multiple sources, including OLS and a subset of mappings provided by the UMLS (Unified Medical Language System). The service is still under development, and users are encouraged to provide feedback. OxO allows users to search for mappings using specific identifiers, or to view all mappings between different (meta)data sources.

The Metadata Schema and Crosswalk Registry (MSCR)^69^ and the Data Type Registry (DTR) of the FAIRCORE4EOSC project are registries for managing and sharing metadata schemata and crosswalks respectively data types within the EOSC ecosystem. They allow users to search, browse, and download metadata schemata and crosswalks or data types via a GUI or an API. Registered users and communities can create, register, and version metadata schemata and crosswalks or data types with GUPRIs.

The Data Repository Service (DRS) API provides a generic interface to data repositories so data consumers, including workflow systems, can access data objects in a single, standard way regardless of where they are stored and how they are managed. The main purpose of DRS is to map a logical ID to a method for retrieving the data represented by the ID.

FAIRsharing.org^70^ is a community-driven platform, with a diverse set of stakeholders from academia, industry, funding agencies, standards organizations, and infrastructure providers. Its goal is to provide enhanced guidance for users of standards, databases, repositories, and data policies, while also increasing producer satisfaction by improving the visibility, reuse, adoption, and citation of resources.

Mapping Commons^61^ is an idea developed and promoted by the Monarch Initiative^71^ and the SSSOM Developer Community, which involves the creation and maintenance of domain-focused, community-curated mapping registries. A template system has been developed to support setting up and maintaining registries, with some support for managing the mapping life-cycle (in particular data model validation).

In addition to considering this existing and ongoing work, it is necessary to strengthen, encourage, and properly acknowledge the collaboration between the associated international stakeholders of FAIR initiatives and projects. This collaboration should be open-source based. Despite the inherent decentralization of the IFDS, we believe that institutions such as libraries, by functioning as trusted focal points within the IFDS, can facilitate and ensure the security of FAIRness. This would entail the aggregation of all three FAIR Service components and the periodic harvesting of (meta)data from other IFDS nodes to index, archive, and serve relevant FDOs as a common good for both humans and machines.

### Concluding remarks

In this paper, we initiated our investigation under the assumption that the FAIR Guiding Principles were conceived in the spirit of LOD and thus inherited the same issues from LOD regarding semantic interoperability. These issues stem from the use of simplistic vocabularies and the lack of foundational (semantic) schemata. These factors pose a major obstacle to the reusability and integration of (meta)data across the Web. We adopted a linguistic perspective on semantic interoperability by examining various aspects underlying the semantic interoperability of English as a natural language. We identified parallels between (i) the structures and conventions that enable the semantic interoperability of terms and statements as two basic types of textual representational artifacts and (ii) the structures of (meta)data schemata. We highlighted the potential for enhancement of the semantic interoperability of the latter. The analysis yielded a conceptual model of semantic interoperability, distinguishing between terminological and propositional interoperability and their subcategories (Fig. 5). This conceptual model subsequently served as the basis for suggesting additional criteria for the FAIR Guiding Principles, extending them towards FAIR 2.0. The study concluded with a concise overview of how FAIR Services contribute to the development of a comprehensive interoperability framework. This framework includes a readily usable interoperability assessment model that not only assesses the FAIRness of basic metadata but also the FAIRness of all kinds of domain-specific (meta)data.

It is the objective of this paper, to contribute to the realization of the IFDS’s objectives of providing an Internet of FAIR (meta)data and services by increasing the overall machine-actionability of (meta)data. We acknowledge that an IFDS cannot be fully interoperable across all its contents. Nevertheless, we anticipate that it will provide all the information necessary to discover all datasets relevant for a given purpose and that it will indicate their cross-interoperability and their interoperability with relevant tools and methods for interacting with them. Such an IFDS would efficiently harness machine assistance to help humans manage the large amounts of (meta)data available, supporting them in sharing, discovering, integrating, and reusing (meta)data across the Web to gather meaningful insights.

## Data Availability

The authors declare they did not use any data in the creation of this article. Therefore, no data must be made available.

## Code availability

The authors declare that no custom code was used in the creation of this article. Therefore, no code must be made available.
